# Highly efficient and low-mosaicism *piggyBac* transgenesis platform for rapid founder phenotyping

**DOI:** 10.1016/j.isci.2026.116648

**Published:** 2026-07-06

**Authors:** Eiichi Okamura, Shoma Matsumoto, Eiji Mizutani, Kazuya Murata, Yoko Tanimoto, Tra Thi Huong Dinh, Hayate Suzuki, Akihiro Kuno, Woojin Kang, Natsuki Mikami, Tomoka Ema, Kento Morimoto, Kanako Kato, Tomoko Matsumoto, Nanami Masuyama, Yusuke Kijima, Genta Nagae, Masanaga Muto, Toshifumi Morimura, Hideto Mori, Fumihiro Sugiyama, Satoru Takahashi, Hiroyuki Aburatani, Knut Woltjen, Nozomu Yachie, Seiya Mizuno, Masatsugu Ema

**Affiliations:** 1 Department of Stem Cells and Human Disease Models, Research Center for Animal Life Science, Shiga University of Medical Science, Otsu, Shiga, Japan; 2 Laboratory of Stem Cell Therapy, Faculty of Medicine, University of Tsukuba, Tsukuba, Ibaraki, Japan; 3 Laboratory Animal Resource Center, Transborder Medical Research Center, University of Tsukuba, Tsukuba, Ibaraki, Japan; 4 Center for One Medicine Innovative Translational Research (COMIT), Institute for Advanced Study, Gifu University, Gifu, Japan; 5 Department of Anatomy and Embryology, Institute of Medicine, University of Tsukuba, Tsukuba, Ibaraki, Japan; 6 Tsukuba Institute for Advanced Research (TIAR), University of Tsukuba, Tsukuba, Ibaraki, Japan; 7 Research Fellow of the Japan Society for the Promotion of Science, Chiyoda-ku, Tokyo, Japan; 8 Ph.D. Program in Human Biology, School of Integrative and Global Majors, University of Tsukuba, Tsukuba, Ibaraki, Japan; 9 Doctoral Program in Medical Sciences, Graduate School of Comprehensive Human Sciences, University of Tsukuba, Tsukuba, Ibaraki, Japan; 10 Department of Life Science Frontiers, Center for iPS Cell Research and Application (CiRA), Kyoto University, Sakyo-ku, Kyoto, Japan; 11 School of Biomedical Engineering, Faculty of Applied Science and Faculty of Medicine, The University of British Columbia, Vancouver, BC, Canada; 12 Institute for Advanced Biosciences, Keio University, Tsuruoka, Yamagata, Japan; 13 Graduate School of Media and Governance, Keio University, Fujisawa, Kanagawa, Japan; 14 Genome Science and Medicine Laboratory, Research Center for Advanced Science and Technology, The University of Tokyo, Meguro-ku, Tokyo, Japan; 15 Premium Research Institute for Human Metaverse Medicine (WPI-PRIMe), The University of Osaka, Suita, Osaka, Japan; 16 International Institute for Integrative Sleep Medicine (IIIS), Life Science Center (TARA), University of Tsukuba, Tsukuba, Ibaraki, Japan; 17 Research Center for Advanced Science and Technology, The University of Tokyo, Meguro-ku, Tokyo, Japan; 18 Institute for the Advanced Study of Human Biology (WPI-ASHBi), Kyoto University, Kyoto, Japan

**Keywords:** transgenic, piggyBac, transposon, conditional knockout

## Abstract

Although pronuclear microinjection is the most widely used method for producing transgenic (Tg) animals, phenotypic characterization is usually performed from the next generation onwards because the production efficiency is limited. Conventional Cre-*lox*P-based conditional knockout (cKO) mouse production requires generating two genetically modified strains and multiple rounds of breeding before cKO mice are available for analysis. Here, we optimized a *piggyBac* transposon-based method of Tg mouse production and established conditions under which nearly all F_0_ embryos are Tg. Using a single-cell RNA sequencing-based strategy, we characterized mosaicism in F_0_ embryos and demonstrated that *piggyBac*-mediated transgene integration occurs early in embryonic development. We also achieved ∼70% efficiency in generating bacterial-artificial-chromosome-Tg mice. By combining this method with genome editing, we developed a strategy for tissue-specific-knockout phenotyping in the F_0_ generation. This platform expands experimental options for Tg animal production by supporting rapid F0-based phenotypic assessment and efficient founder generation for subsequent breeding.

## Highlights

• Optimized *piggyBac* transgenesis yields nearly all transgenic F0 embryos
• BAC transgenic mice are generated with approximately 70% efficiency
• DNA barcoding and scRNA-seq enable assessment of founder mosaicism
• ScKiP enables rapid F0-based conditional knockout phenotyping

## Graphical abstract

**Figure:**
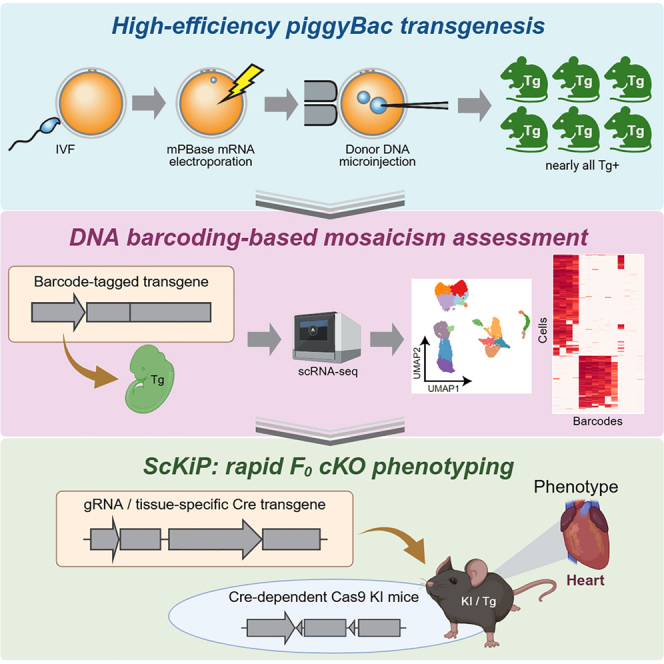

## Teaser

therapeutic procedure; experimental models in systems biology; biological sciences research methodologies

## Introduction

Transgenic (Tg) technology enables the introduction of foreign DNA sequences—either from other species or artificially designed—into the genomes of organisms. It has been widely applied to generate organisms with desirable traits, such as resistance to infectious diseases, enhanced growth, and herbicide tolerance. In the medical field, Tg technology has contributed greatly through the generation of disease model animals and the elucidation of gene function.^1^^,^^2^^,^^3^^,^^4^ Another approach for inserting foreign DNA is the knockin (KI) method, which targets specific loci, in contrast to the random integration achieved with Tg technology. Although KI allows more predictable genetic modifications, it is generally less efficient and has difficulty introducing large DNA fragments. While recent advances in genome editing have improved KI efficiency in some animals, such as mice and rats,^5^^,^^6^^,^^7^ there is still a reliance on Tg technology, especially for large animal species—including rabbit, pig, and cattle—for which efficient KI systems have yet to be established.

Jaenisch and Mintz reported the first Tg animal in the 1970s, in which a retrovirus was employed as the vector for producing the mouse.^8^ In 1980, a pronuclear (PN) microinjection method, which remains the most widely used for producing Tg mice, was reported.^9^ Despite its popularity, this approach achieves a success rate of only ∼20% at best.^10^ As a result, sufficient Tg mice are rarely obtained in the founder (F_0_) generation, and phenotypic analyses on subsequent generations are usually necessary. Improving the efficiency of Tg production is therefore essential, both to accelerate research and to reduce the use of animals in accordance with animal welfare principles. A more efficient approach, lentivirus-mediated transgenesis, was introduced in 2003,^11^ enabling Tg mouse generation with efficiency in the range of 80%–100%. However, this method is limited by the maximum insert size of ∼8 kbp.

Another method for generating Tg animals is the transposon-based approach. Transposons are mobile DNA sequences that can move within the genome and were first discovered in maize.^12^ During transposition, transposase enzymes bind to the inverted terminal repeats (ITRs) that define the boundaries of the transposon and cut out the DNA sequence between ITRs from the genome and insert it into a new genomic location. Several transposon systems have been developed and applied to produce Tg animals, including *Tol2*,^13^ *Sleeping Beauty*,^14^ and the *piggyBac*^15^ system. These methods can introduce large transgenes in excess of 100 kbp; however, their efficiency is generally below 70%, which is lower than that of the lentiviral method.

Recent advances in genome editing technology now enable the efficient production of systemic knockout (KO) animals, allowing large-scale phenotypic screening at the F_0_ generation.^16^ However, approximately 23% of gene KOs result in embryonic lethality,^17^ necessitating tissue-specific KOs to investigate gene functions in a particular tissue. Moreover, the establishment of tissue-specific Cre Tg and flox mouse strains is time-consuming and labor-intensive. Although many such strains have been generated and valuable bioresources established, tissue-specific KO typically requires more than a year to obtain, cross, and generate the next generation for phenotypic analysis. To address this issue, various methods have been developed to achieve tissue-specific KO in a single generation,^18^^,^^19^^,^^20^^,^^21^^,^^22^^,^^23^^,^^24^ but each method has distinct advantages and limitations, and the selection of one should be based on the particular purpose of the research. In the present study, we evaluated an approach by electroporating mPBase mRNA immediately after fertilization and subsequently introducing donor DNA into the pronucleus, with the aim of synchronizing transposase availability with the timing of donor DNA delivery to enhance transgenesis efficiency and suppress F_0_ mosaicism. As a result, we achieved nearly 100% efficiency in producing Tg mice. Using a single-cell RNA sequencing (scRNA-seq)-based approach, we characterized the degree and pattern of mosaicism in F_0_ embryos and showed that *piggyBac*-mediated transgene integration occurs predominantly early in embryonic development. This method also enabled efficient introduction of an approximately 170 kbp bacterial artificial chromosome (BAC) vector with ∼70% efficiency. We also report a strategy for generating tissue-specific conditional KO, termed ScKiP (single-step cKO mouse production with *piggyBac*), by leveraging our highly efficient, low-mosaicism *piggyBac* transgenesis approach. Unlike conventional methods that require lengthy breeding of floxed and Cre-Tg lines, ScKiP allows rapid, single-generation phenotyping of essential genes. Thus, ScKiP has the potential to provide a useful framework for functional genomics while also helping to reduce the number of animals required, thereby contributing to both scientific advancement and animal welfare.

## Results

### Optimization of *piggyBac* transgenesis by electroporation and PN microinjection

**Figure 1:**
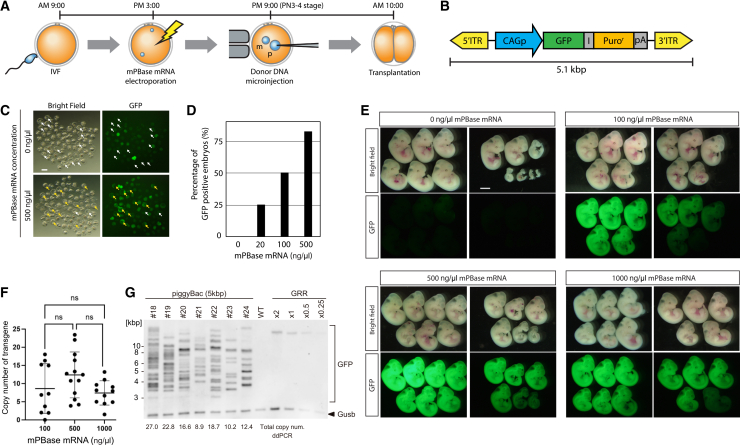
Optimization of Tg generation by *piggyBac* (A) Schematic representation of the method to introduce mPBase mRNA and donor plasmid DNA. (B) Schematic representation of CAG-EGFP-IRES-Puro donor plasmid DNA. (C and D) Optimal concentration of mPBase mRNA to generate GFP-positive blastocysts. Fluorescence microscopic images of conditions with mRNA at 0 and 500 ng/μL are shown in (C). Weak GFP signals observed in the absence of mPBase (0 ng/μL) represent background signals arising from transposase-independent random integration and/or transient expression of the donor DNA. Tg embryos were judged by GFP intensity above background (orange arrow: GFP-positive blastocysts, white arrow: background-level/GFP-negative blastocysts). Embryos without arrows were developmentally arrested and excluded from quantitative evaluation. GFP-positive rates are displayed as a bar graph in (D). Scale bars, 100 μm. (E) Embryos at E11.5 generated by various concentrations of *piggyBac*. Scale bars, 2 mm. (F) Transgene copy number in embryos generated under various doses (100, 500, 1,000 ng/μL) of mPBase mRNA. The data are presented as the mean ± values SD. Statistical analyses were performed using ordinary one-way ANOVA. ns, not significant (p ≥ 0.05). (G) Southern blot analysis of DNA from the E11.5 transgenic embryos shown in (E). The number shown at the top of the blot is the copy number measured by ddPCR analysis. *GRR* mouse was used as a single copy control.

To establish a clear benchmark for *piggyBac*-mediated transgenesis in mice, we first performed an extensive literature search covering *piggyBac* variants (PBase, mPBase,^25^ and hyPBase^26^: the original *piggyBac* transposase and engineered variants with improved activity), delivery formats (plasmid versus mRNA), injection strategies, and mouse strains (Table S1). This systematic survey revealed that overall Tg efficiency has not improved substantially across the literature over the past two decades, and many studies still report only modest efficiencies. Moreover, many reports do not rigorously evaluate F_0_ mosaicism. Importantly, delayed onset of transposase activity relative to donor DNA delivery may reduce Tg efficiency and increase F_0_ mosaicism by shifting integration to later developmental stages. We therefore reasoned that earlier availability of transposase immediately after fertilization would improve F_0_ efficiency and reduce mosaicism, and hypothesized that efficiency could be improved further by initiating the expression of mammalian codon-optimized mPBase earlier than in previous methods (Figure 1A). To test this, shortly after *in vitro* fertilization, we electroporated various concentrations of mPBase mRNA (0, 20, 100, and 500 ng/μL) into zygotes, followed by PN injection of the donor plasmid CAG-GFP (CAG, CMV immediate-early enhancer/chicken β-actin promoter; Figure 1B) at the PN 3–4 stage (Figure 1A). The embryos were cultured to the blastocyst stage and GFP-positive blastocysts were counted (Figure 1C). The highest rate (∼80%) was obtained with 500 ng/μL (Figure 1D). To confirm this finding at a later stage, we transplanted two-cell-stage embryos into surrogate mothers. Most E11.5 embryos were GFP-positive as determined by fluorescence microscopy (Figure 1E), and droplet digital PCR (ddPCR) analysis showed that 9 of 10 were Tg in the 100 ng/μL group, while all embryos were Tg in the 500 and 1,000 ng/μL groups (Figure 1F). The 500 ng/μL group showed the highest mean copy number (Figure 1F), suggesting that 500 ng/μL mPBase mRNA is optimal for producing Tg mice.

To further support the generality of the gene-delivery framework used in this study, we examined whether substituting hyPBase for mPBase would yield comparable performance under otherwise identical conditions. After determining the optimal concentration of hyPBase at the blastocyst stage (Figures S1A–S1C; Table S2), embryo transfer experiments showed similarly high Tg rates for hyPBase and mPBase (hyPBase: 83%; mPBase: 69%) (Figures S1D and S1E; Table S2). In addition, transgene copy-number distributions assessed by ddPCR and southern blotting were comparable between the two conditions (Figures S1F and S1G). These results indicate that the high-efficiency outcome is not only restricted to mPBase but is also observed when hyPBase is used.

However, despite these high efficiencies under both mPBase and hyPBase conditions, southern blot analysis revealed more bands than expected from ddPCR in some embryos, suggesting that integration events occur after the onset of DNA synthesis in fertilized embryos and generate cell populations with distinct integration sites, consistent with F_0_ mosaicism (Figures 1G and S1G).

### Timing and site of the Tg integration by PN microinjection

**Figure 2:**
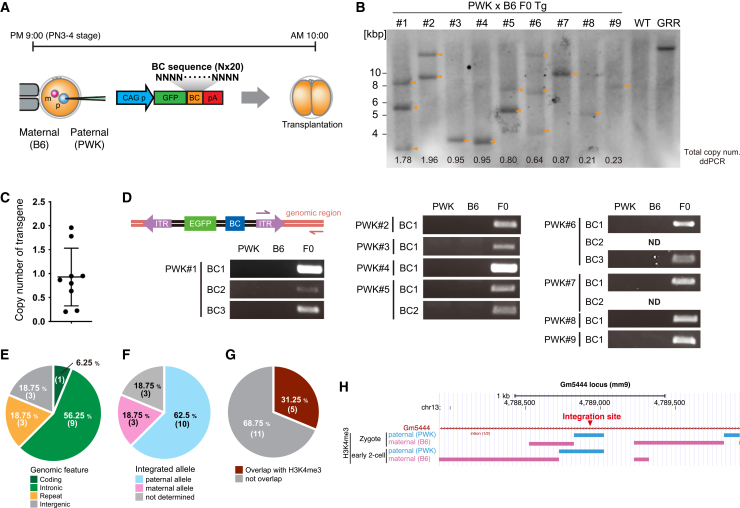
Preferential integration of transgene into paternal genome after Tg DNA injection into paternal nucleus (A) Schematic showing the timing of donor DNA microinjections relative to cell-cycle progression in the mouse embryo. (B) Southern blotting for GFP in E11.5 Tg embryos. WT mouse and *GRR* KI mouse were used as negative and single copy controls, respectively. (C) Total copy number of E11.5 Tg embryos by ddPCR. The data are presented as the mean ± values SD. (D) Inverse PCR to identify genomic integration site for Tg. (E) Summary of genome-wide distribution of the transgene integration sites. (F) Summary of the transgene integration allele. (G) Percentage of transgene insertion sites overlapping with H3K4me3 peaks. (H) An example of a transgene integration site overlapping with H3K4me3 peaks.

To investigate the timing and genomic sites targeted by our optimized *piggyBac* system, we used eggs fertilized with PWK/Phj (PWK) strain sperm and C57BL/6J (B6) strain oocytes, allowing us to distinguish integration events on paternal and maternal alleles (Figure 2A). After electroporation of mPBase, followed by PN injection of a donor plasmid containing GFP and a DNA barcode (BC) sequence, the fertilized eggs were transferred into surrogate mothers next day at the two-cell stage. Among 17 E11.5 embryos, 9 were identified as Tg by fluorescence microscopy and genomic PCR (Figures S2A and S2B). Southern blotting revealed that each Tg embryo carried one to three integrations at distinct genomic loci (Figure 2B). ddPCR analysis further indicated that five Tg embryos had copy numbers of less than one (Figure 2C), indicating integration events occurred after DNA synthesis began. To map the genomic integration sites, we performed inverse PCR and confirmed the results by genotyping PCR using BC-specific primers (Figure 2D). Of the 16 integration events analyzed, 9 were located in introns, 3 in repeat sequences, 3 in intergenic regions, and 1 within a coding sequence (Figure 2E). Using SNPs between the PWK and B6 genomes, we determined that 10 of the 13 integrations occurred on the paternal allele, while the remaining 3 integrations occurred on the maternal allele (Figure 2F). This suggested that mPBase-mediated integration is initiated in the injected male pronucleus but continues after PN fusion. To further characterize integration site preference, we performed a comparative analysis using chromatin immunoprecipitation sequencing (ChIP-seq) data. Previous reports indicated that the integration sites are enriched in regions marked by H3K4me3.^27^ Consistent with this, comparison with publicly available ChIP-seq peaks^28^ showed that 31.25% of our integration sites overlapped with H3K4me3-enriched regions (Figures 2G and 2H).

### *piggyBac* transgenesis by ICSI-Tr method

Mosaicism was observed when donor DNA was introduced at the PN stage (Figures 1 and 2), coinciding with the onset of DNA synthesis, and therefore we tested whether introducing donor DNA earlier (i.e., before the PN stage) could enable transgene integration prior to DNA replication. Previous studies reported that intracytoplasmic sperm injection (ICSI)-Tr, in which damaged sperm are co-injected with foreign DNA, yielded ∼20% Tg efficiency,^29^ which was later improved to ∼40% using plasmid-based mPBase and donor DNA.^30^^,^^31^ However, with fresh sperm and the *piggyBac* system, efficiency was only 18.8%.^31^ We hypothesized that replacing plasmid DNA with mPBase mRNA could further improve efficiency and therefore optimize a modified version of the ICSI-Tr method. We first injected mRNA into unfertilized eggs, treated them with strontium chloride to improve survival,^32^ and subsequently injected fresh sperm with donor plasmid DNA (Figure S3A). To determine the optimal donor DNA concentration, we fixed mPBase mRNA at 100 ng/μL and tested five donor concentrations (0–50 ng/μL) carrying CAG-GFP (Figure S3B). After ICSI, eggs were cultured to the two-cell stage, transferred to surrogate mothers, and recovered at E11.5 (Figure S3C). The proportion of GFP-positive embryos increased with donor DNA concentration, but survival declined at 50 ng/μL (Figure S3D; Table S3). Using the optimal donor DNA concentration, we then tested six mPBase mRNA concentrations (1–500 ng/μL). The percentage of GFP-positive embryos and mean copy number as determined by ddPCR did not differ significantly among these concentrations, although the median peaked at 5 ng/μL (Figures S3E and S3F; Table S3). We next evaluated the effects of strontium chloride and sperm freeze-thawing (Figure S3G). With fresh sperm and strontium chloride, GFP-positive embryos were obtained at 75% efficiency, which was largely unchanged with frozen-thawed sperm. In contrast, the omission of strontium chloride reduced the efficiency to 34.8% (Figure S3H and Table S3). Thus, using mPBase mRNA enabled efficient ICSI-Tr with fresh sperm, although it remained slightly lower than that of electroporation and PN injection. Notably, southern blot analysis consistently detected more bands than expected from ddPCR (Figure S3I) and the ddPCR value was below 1 in ∼20% embryos, indicating mosaicism under this method.

### scRNA-seq with DNA barcoding identifies early stage integration and mosaicism

The *piggyBac* systems established in this study revealed evidence of F_0_ mosaicism in individual embryos (Figures 1 and 2; Figures S1 and S3). To assess the degree and pattern of mosaicism in F_0_ embryos, we initially developed a method to detect BCs integrated into the genome because some BCs may be silenced or lost and artifacts could arise during the PCR and next-generation sequencing (NGS) procedures. To address this, cloned mouse embryonic stem cell (mESC) lines carrying multiple BC-linked transgene cassettes (Figures S4A and S4B) were analyzed by amplicon sequencing (amplicon-seq) (Figure S4C). We hypothesized that *bona fide* BCs would be detected as significant read counts, accompanied by a sharp peak of quotient (Figure S4D). Accordingly, 20 and 9 BCs were identified in #6–10 and #13–25 ESCs, respectively (Figure S4D). To validate them, BC-specific genotyping for individual transgenes was performed (Figure S4E), confirming all detected BCs (Figures S4F and S4G). The number of BCs estimated by amplicon-seq closely matched that determined by ddPCR (Figure S4H), indicating that this method reliably detects *bona fide* genomic BCs. To assess the sensitivity, we mixed genomic DNA from the two mESC lines at ratios of 1:1, 1:3, and 1:7, performed amplicon-seq, and applied this method (Figure S4I). All 29 BCs were detected in these mixtures (Figure S4J), demonstrating that our system can reliably identify mosaicism arising from integration and/or excision events occurring as late as the eight-cell stage.

**Figure 3:**
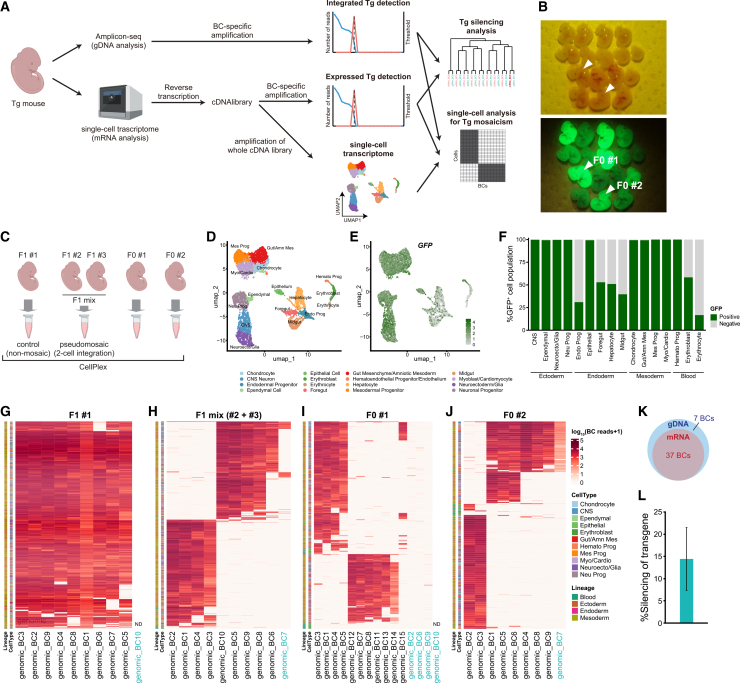
Identification of integration timing of Tg DNA by scRNA-seq analysis with DNA barcoding (A) A flow chart of the mosaicism analyses. From the single-cell suspension of Tg mice, both bulk genome and single-cell transcriptome libraries were constructed. A single-cell cDNA library was used for conventional scRNA-seq library and BC-specific library construction. (B) Fluorescent images of two F_0_ embryos. Bright field (left) and GFP fluorescence (right) are shown. (C) UMAP of mouse embryos with cell-type annotation. Each color represents cell type. (D) The expression levels of GFP in each cell. (E) The GFP-positive ratio for each cell type. (F) Evaluation of integrated transgenes by single-cell resolution using BC vector. F_1_ #1 and an artificial mosaic control sample (“F_1_ mix”), generated by experimentally mixing cells from two distinct F1 embryos (F_1_ #2 and F_1_ #3), were used as control. F_1_, F_1_ mix, and two F_0_ samples (F_0_ #1 and #2) were multiplexed using the CellPlex technique before the construction of an scRNA-seq library. (G–J) A heatmap shows the expression levels of individual transgenes integrated into the genome for (G) F_1_ #1, (H) F_1_ mix (artificial mosaic control), and (I, J) F_0_ mice. The expression of each transgene was distinguished by the BC sequence. Cell type and lineage are shown by the color bar on the left of the heatmap. The transgenes highlighted in blue were silenced. (K) Venn diagram for genomic and transcribed transgenes. Seven BCs were only identified by genomic analysis. (L) The ratio of transgene silencing by position-effect variegation. The bar graph shows mean value (±SD). Parts of this figure were created in BioRender. Okamura, E. (2026) https://BioRender.com/6lepdkn.

On the basis of this method to analyze genomic BCs, we next devised a scRNA-seq-based strategy combined with a DNA BC-linked transgene cassette to assess the degree and pattern of mosaicism in F_0_ embryos (Figure 3A). Fertilized eggs were electroporated with mPBase mRNA and microinjected with donor DNA carrying an EGFP cassette linked to DNA BC sequence. At E11.5, Tg embryos were identified by fluorescence (Figure 3B) and subjected to scRNA-seq with CellPlex technology (Figure 3C). A total of 5,745 cells from Tg samples (*n* = 4) were analyzed (Figure S5A), comprising two F_0_ Tg embryos (F_0_ #1 and #2), one F_1_ Tg embryo (F_1_ #1), and an artificial mosaic control sample (“F_1_ mix”) generated by experimentally mixing cells derived from two distinct F_1_ Tg embryos (F_1_ #2 and F1 #3). Uniform manifold approximation and projection (UMAP) clustering revealed 16 cell types (Figure 3D; Figures S5B–S5G). EGFP transcripts were detected in a substantial proportion of these cell types (Figures 3E and 3F). Subsequently, we analyzed genomic BCs in the two F_0_ embryos (F_0_ #1 and #2) as well as in the F1 control (F_1_ #1) and the artificial mosaic control (“F_1_ mix”) (Figure 3C). We identified 10 genomic BCs in F_1_ #1, 4 in F_1_ #2, 6 in F_1_ #3, 15 in F_0_ #1, and 9 in F_0_ #2 embryos (Figure S6A). Transcribed BCs were then detected by scRNA-seq, assuming that *bona fide* transcripts would appear as significant read counts, as in genomic BCs (Figure S6B). All transcribed BCs matched their genomic counterparts, whereas several genomic BCs were not detected as RNA, indicating transcriptional silencing (Figure S6C). In F_1_ #1, scRNA-seq revealed that all nine transcribed BCs were expressed nearly ubiquitously across the cell types, but weakly in several cell types including hepatocytes, erythrocytes, and foregut, midgut, and endoderm progenitors, reflecting low CAG promoter activity in these lineages. These cell types were thus excluded from further analysis (Figures 3G, S5D, and S5E). Consistent with its design as a positive control for detecting mosaic states, individual BCs in the F1 mix sample were enriched exclusively in cell clusters derived from one or the other F_1_ embryo (Figure 3H), supporting the ability of our framework to detect mosaic compositions. Notably, in two F_0_ embryos, we observed two major clusters spanning multiple tissues, consistent with integration occurring predominantly at the two-cell stage (Figures 3I and 3J). Overall, 14.4% (7/44) of the integrated transgenes appeared to be silenced, possibly due to position-effect variegation (Figures 3K and 3L). These findings indicate that, under our optimized protocol, *piggyBac*-mediated transgene integration occurs predominantly early in embryogenesis, while residual founder mosaicism remains detectable.

### Efficient integration of large DNA fragments by *piggyBac* transposase

**Figure 4:**
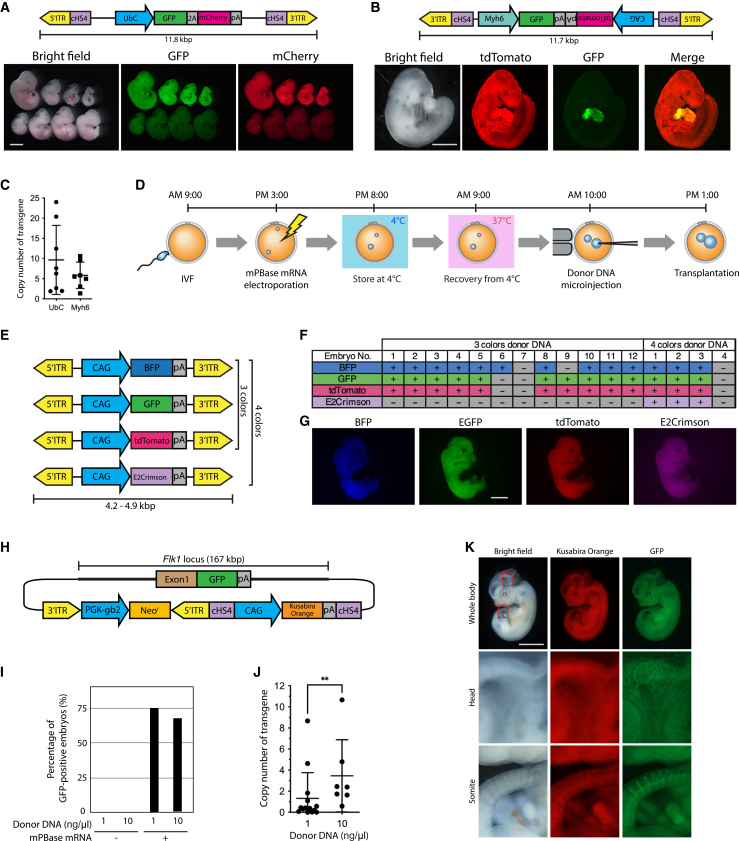
Integration of large DNA fragments by *piggyBac* (A) Fluorescent images of E11.5 embryos harboring UbC pro.-GFP-P2A-mCherry transgene. cHS4: chicken hypersensitive site-4 insulator element. Bright field, GFP fluorescence, and mCherry fluorescence images are shown. (B) Fluorescent images of representative E9.5 embryos harboring Myh6 pro.-GFP-CAG-tdTomato transgene. Bright field (left) and tdTomato fluorescence images (right) are shown. Scale bars, 100 μm at E9.5. (C) Copy number analysis of Tg embryos harboring UbC pro. GFP-P2A-mCherry transgene (UbC) and Myh6 pro. GFP-CAG-tdTomato transgene (Myh6). The data are presented as the mean ± values SD. (D) Schematic representation of the method to introduce mPBase mRNA and donor plasmid DNA. Fertilized eggs were temporarily refrigerated after electroporation. (E) Schematic representation of BFP, EGFP, tdTomato, and E2Crimson expression cassettes on the donor plasmids. (F) Summary of the results of observing the obtained embryos by fluorescence microscopy. The presence and absence of each fluorescence color are indicated by + and −, respectively. The results of three- (top) and four-color donor plasmid injection (bottom) are shown separately. (G) Fluorescent images of representative E11.5 embryo harboring four-color transgenes. BFP, EGFP, tdTomato, and E2Crimson fluorescent images are shown. (H) Schematic representation of BAC donor vector. (I) Optimization of the concentration of donor DNA using DNAs from E11.5 embryos. Transgenes were detected by conventional PCR and GFP-positive rates are displayed as a bar graph. Blue dots indicate E11.5 development rates for the number of transplanted two-cell-stage embryos. (J) Copy number analysis of BAC Tg. Transgene copy numbers were determined by ddPCR using DNA from E11.5 embryos. The data are presented as the mean ± values SD. Statistical analyses were performed using Mann-Whitney test. ∗∗p ≤ 0.01. (K) Fluorescent images of a representative E11.5 BAC Tg embryo. Bright field, Kusabira-Orange fluorescence, and GFP fluorescence images of the whole body, head, and somite region are shown. The red rectangular frames inside the whole body represent the head and somite region. Scale bars, 2 mm at E11.5.

Relatively short plasmids were used as the donor DNA in our assay; therefore, we next tested larger donor DNAs carrying multiple expression cassettes. We constructed two plasmids containing a 12 kbp transgene. One construct carried a GFP-P2A-mCherry cassette under the Ubiquitin C promoter (Figure 4A). All eight E11.5 embryos exhibited GFP and mCherry expression (Figure 4A; Table S4). The other construct carried GFP under the *Myh6* promoter and tdTomato under the CAG promoter (Figure 4B). Heart-specific GFP expression was confirmed in all E9.5 embryos by fluorescence microscopy (Figure 4B; Table S4). ddPCR revealed mean copy numbers of 9.7 ± 8.6 and 5.8 ± 3.3 for the UbC-GFP-P2A-mCherry and Myh6-GFP-CAG-tdTomato constructs, respectively (Figure 4C).

Although the electroporation-microinjection method developed in this study yields Tg mice with very high efficiency, its experimental schedule is inconvenient because microinjection must be performed late at night. To address this, we modified the protocol by storing eggs after electroporation in a refrigerator and performing microinjection the following morning (Figure 4D). After microinjecting either a mixture of BFP/EGFP/tdTomato expression vectors (three colors) or a mixture of BFP/EGFP/tdTomato/E2Crimson (four colors) (Figure 4F), the fertilized eggs were transferred into surrogate mothers at the one-cell stage. The embryos were dissected at E14.5, and fluorescence was checked as an indicator of Tg. Under the three-color conditions, 11 of 12 embryos (91.7%) were Tg, and under the four-color conditions, 3 of 4 embryos (75%) were Tg (Figure 4G). These results demonstrate that Tg mice can be generated at high efficiency even after refrigerated storage of the fertilized eggs. Notably, this refrigerated storage step is optional and serves primarily to improve scheduling flexibility.

The 12 kbp Tg mice were established with 100% efficiency, and therefore we next applied this method to generate BAC Tg mice because they are typically produced with very low efficiency. In a previous study, we constructed BAC DNA carrying GFP driven by the *Flk1* promoter.^33^ For this study, ITR sequences and a Kusabira-Orange cassette were inserted into the BAC vector backbone (Figure 4H). The engineered BAC DNA was tested as a donor DNA at 1 or 10 ng/μL. Owing to high viscosity, concentrations above 10 ng/μL were not feasible. At 1 ng/μL, 14 of 19 E11.5 embryos were Tg, while at 10 ng/μL, 7 of 10 embryos were Tg only when mPBase was introduced (Figure 4I). The overall Tg efficiency did not differ significantly between concentrations. However, ddPCR revealed significantly different mean copy numbers of 0.39 and 2.39 for 1 and 10 ng/μL, respectively (Figure 4J). BAC Tg embryos showed ubiquitous Kusabira-Orange expression and GFP signals in blood vessels (Figure 4K), consistent with previous findings.^32^ PCR genotyping further indicated that Tg transmission was efficient, with 52 of 94 offspring (55%) being Tg.

### Rapid phenotyping of conditional KO mice at the F_0_ generation by ScKiP

**Figure 5:**
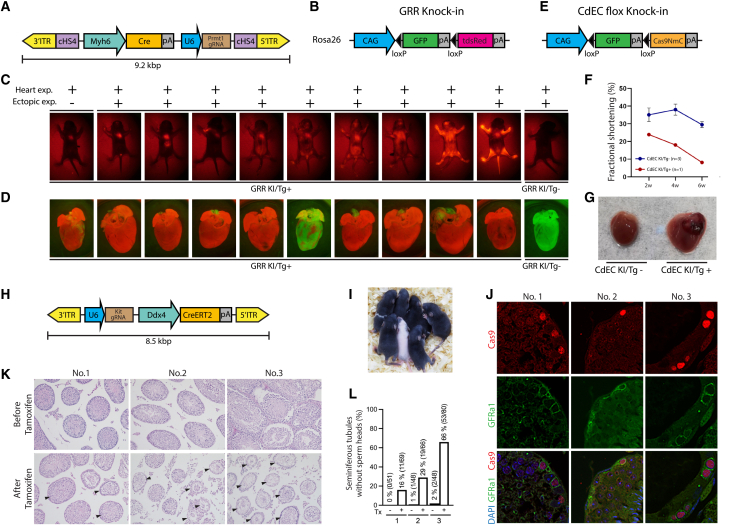
Rapid phenotyping of conditional KO mice (A) Schematic representation of Myh6-Cre-Prmt1 gRNA donor plasmid DNA. Only the region between two ITRs is described. cHS4: chicken hypersensitive site-4 insulator element. pA: polyadenylation signal. (B) Schematic representation of GRR KI allele. (C) Fluorescent images of born GRR KI mice with Myh6-Cre-Prmt1 gRNA transgene (GRR KI/Tg+) at the P4 (postnatal day 4) stage. Fluorescent image of GRR KI mice without the transgene (GRR KI/Tg−) is shown as a control. (D) Fluorescent images of heart from GRR KI/Tg+ and GRR KI/Tg− mice. (E) Schematic representation of CdEC flox KI allele. (F) Results of echocardiography on 2-, 4-, and 6-week-old CdEC flox KI mouse with the Myh6-Cre-Prmt1 gRNA transgene (CdEC KI/Tg+) or without it (CdEC KI/Tg−). The data are presented as the mean ± SEM. (G) Morphological observation of hearts from 6-week-old CdEC KI/Tg+ and CdEC KI/Tg− mice. (H) Schematic representation of gRNA-Ddx4-CreERT2 donor plasmid DNA. Only the region between two ITRs is described. (I) Observation of newborn CdEC KI mice with the gRNA-Ddx4-CreERT2 transgene (CdEC KI/Tg+). (J) Results of HE-staining analysis of testes removed from CdEC KI/Tg+ mice before and after tamoxifen injection. Arrowheads indicate seminiferous tubules not containing sperm heads. (K) Percentage of seminiferous tubules without sperm heads in the testes from CdEC KI/Tg+ mice before and after tamoxifen injection. (L) Results of the immunostaining analysis of testes from CdEC KI/Tg+ mice after tamoxifen injection using anti-Cas9 and anti-GFRα1 antibodies.

Phenotypic analysis of essential genes in a certain tissue requires use of the Cre-loxP system, which is time-consuming and labor-intensive. Using the method developed in this study, we established a strategy to generate cKO mice directly at the F_0_ generation via *piggyBac* transgenes. We first targeted *Prmt1*, whose systemic KO is embryonically lethal but whose heart-specific KO causes dilated cardiomyopathy in young mice.^34^ For heart-specific KO, we constructed a donor plasmid with Cre under the cardiac-specific *Myh6* promoter and a gRNA targeting *Prmt1* (Figure 5A). Before the cKO experiment, Cre activity was verified using a GRR mouse,^35^ a green-to-red fluorescent-convertible Cre-reporter strain, in which tdsRed was expressed following Cre-mediated excision (Figure 5B). After PN injection of the donor plasmid into fertilized eggs stored overnight at 4°C, 14 offspring were obtained, 10 of which carried the transgene (GRR KI/Tg+). All showed strong tdsRed expression in the heart, with some ectopic signals including the skin (Figures 5C and 5D). To generate heart-specific *Prmt1* KO, we used *CdEC flox* mice, formally designated C57BL/6J-Gt(ROSA)26Sor<em1(floxed-EGFP-Cas9NmC)Utr>, carrying a floxed Cas9NmC in which nuclear localization signal (NLS)-tagged Cas9 was expressed only after Cre expression (Figure 5E). *Myh6-Cre* donor DNA was injected into fertilized eggs from a CdEC flox mouse strain, and 10 offspring were obtained, all of which had the KI allele but only one had the transgene (CdEC KI/Tg+). Echocardiography revealed impaired cardiac contractility in the CdEC KI/Tg+ mouse compared with that in the control (CdEC KI/Tg−, Figure 5F). At 50 days, the CdEC KI/Tg+ mouse showed signs of heart failure and was euthanized. Its heart weight was nearly double that of the control (188 vs. 101 mg; Figure 5G), consistent with cardiomyopathy reported in conventional cKO mice.^34^

To quantify on-target editing and assess mosaicism, we performed amplicon sequencing of the *Prmt1* target locus using genomic DNA from heart and tail, and analyzed the sequencing reads using CRISPResso2.^36^ While the WT control showed minimal background level variants, the CdEC KI/Tg+ mouse exhibited approximately 20% mutant reads in the heart. Notably, a comparable fraction of mutant reads was also detected in tail DNA, suggesting ectopic Cas9 activation outside the heart lineage (Figure S7). Finally, to address potential off-target mutagenesis arising from sustained Cas9/gRNA activity, we examined all CRISPOR-predicted off-target candidates (*n* = 8) by Sanger sequencing followed by tracking of indels by decomposition (TIDE) analysis^37^ (Figure S8 and Table S5). While Sanger trace-based indel estimation can be variable at low editing frequencies, we independently confirmed that on-target editing estimates obtained by TIDE were comparable to those obtained by amplicon-seq (Figure S7) under our experimental conditions, supporting the use of this workflow for assessment in the present study. Accordingly, low-level apparent off-target signals were interpreted conservatively, consistent with prior benchmarking indicating that detection sensitivity can be limited in the few percent indel range.^38^ Under these criteria, we did not detect clear sequence alterations at the predicted off-target loci. Thus, a *Prmt1* KO phenotype in the heart could be observed at the F_0_ generation, although the rate of Tg mice was low (10%), likely due to ectopic transgene expression causing embryonic lethality.

We next applied this strategy to generate germ cell-specific KO of *Kit* at the F_0_ generation. Hypomorphic *Kit* mutant (*W*^*V*^/*W*^*V*^) lacks sperm, whereas systemic KO (*W*/*W*) causes perinatal- or late fetal-stage lethality.^39^ To avoid lethality from ectopic Cre-loxP recombination, we used germ cell-specific *Ddx4*-CreERT2, which is tamoxifen-inducible, in combination with a gRNA targeting *Kit* (Figure 5H). Following PN injection into fertilized eggs from CdEC flox KI male, 14 donor offspring (11 males, 3 females) were obtained, all carrying both the KI allele and the transgene (Figure 5I). Nine of these males were analyzed. At 16 to 17 weeks old, one testis was removed as a control; tamoxifen was then administered at 18, 22, and 23 weeks. Six mice died, likely due to tamoxifen toxicity, complications, or ectopic *Kit* KO. The remaining three males were euthanized at 32 weeks and their testes were analyzed. HE staining revealed increased seminiferous tubules lacking sperm after tamoxifen compared with the findings before treatment (Figure 5J). We quantified the proportion of Cas9-positive cells (Cas9+/GFRα1+) in three independent animals, yielding values of 8.5% (12/142) for animal #1, 85.7% (108/126) for animal #2, and 59.0% (158/268) for animal #3 (Figure 5K). Notably, the animal exhibiting the mildest phenotype (#1) showed the lowest proportion of Cas9-positive cells, suggesting that cellular-level Cas9 expression is a critical determinant of reliable phenotype detection (Figure 5L). We named this cKO approach “ScKiP,” (single-step cKO mouse production with *piggyBac*), enabling rapid phenotyping of cKO mice at the F_0_ generation.

## Discussion

In this study, we established a highly efficient method for producing Tg mice using the *piggyBac* transposon system. Three transposon systems are commonly used to generate Tg animals: *piggyBac*, *Sleeping Beauty*, and *Tol2*.^40^ We chose *piggyBac* because it is uniquely capable of integrating transgenes larger than 150 kbp.^41^ By combining mPBase mRNA electroporation with PN donor DNA microinjection, we achieved nearly 100% integration efficiency for plasmid-size transgenes and ∼70% efficiency for BAC transgenes.

For conventional Tg studies aimed at establishing germline-transmitted lines, currently available efficiencies may often be sufficient. In contrast, the central goal of the present study is to establish a platform for phenotypic analysis directly in F_0_ animals. From the standpoint of ethical responsibility in animal research, particularly the Reduction principle of the 3Rs, it is important to maximize Tg efficiency and reduce the fraction of transgene-negative F_0_ animals as close to zero as possible.

In this study, we evaluated two distinct nucleic acid delivery strategies: (1) electroporation of *piggyBac* mRNA followed by PN injection of donor DNA, and (2) cytoplasmic mRNA injection combined with ICSI-based co-injection of donor DNA (ICSI-Tr). We optimized the ICSI-Tr method,^31^^,^^42^ substituting mPBase mRNA for plasmid DNA and this approach yielded high efficiency. Notably, strontium chloride treatment improved Tg production, probably by inducing calcium oscillations that activate eggs and promote mRNA translation. Variation of the mRNA concentration had little effect on the efficiency of Tg mouse production, likely because mPBase translation was saturated even at low levels. Nonetheless, Tg efficiency with the ICSI-Tr method remained below 100%, possibly because donor DNA injected into the cytoplasm was diluted or sequestered before it could access the nucleus. Indeed, higher DNA concentrations correlate with better efficiency in Tg production. Because ICSI requires substantial technical expertise and therefore presents a barrier to broad adoption, we position the electroporation-based approach as the standard and broadly accessible protocol of this study, whereas the ICSI-based framework serves as an alternative option. A further simplification of the ICSI-Tr workflow may be to co-inject *piggyBac* mRNA together with donor DNA and sperm. Although this approach is technically plausible, as reported for sperm-mediated genome editing using Cas9 mRNA and gRNA,^43^ its efficiency in the *piggyBac* system would need to be re-evaluated after optimization of mRNA and donor DNA concentrations.

F_0_ mosaicism arises when integration occurs after the first zygotic division. Classical PN microinjection often yields such patterns, with later injections increasing mosaicism.^44^ In earlier studies, mPBase expression plasmids were injected into the cytoplasm of unfertilized eggs or the pronucleus of fertilized eggs.^15^^,^^30^^,^^31^^,^^45^ Urschits et al. showed that mPBase protein expression was negligible for 6 h after injection of the expression plasmid, and peaked ∼30 h post-injection (i.e., after the two-cell stage), by which time donor DNA may be degraded or diluted.^30^ Other studies used mPBase mRNA, but injected it at the PN stage,^46^^,^^47^^,^^48^^,^^49^^,^^50^ which may still delay the onset of transposase activity relative to the timing of donor DNA delivery. Such delays may reduce transgenesis efficiency and increase F_0_ mosaicism by shifting integration to later developmental stages. By delivering mPBase mRNA immediately after fertilization and introducing donor DNA at the PN stage, our *piggyBac* pipeline shifts integration earlier, before notable donor dilution, resulting in low rates of mosaicism while maintaining very high transgenesis efficiency. Further reduction of F_0_ mosaicism may require shortening the effective exposure window of embryos to both transposase activity and donor DNA. In this regard, titrating PBase mRNA to the lowest concentration that maintains sufficient transgenesis efficiency, earlier delivery of both components as attempted in the ICSI-Tr framework, and temporally controllable transposase systems may represent useful future strategies to restrict PBase activity and promote integration before DNA replication. For example, ligand-controllable *piggyBac* transposases, including shld1 (Shield-1)- or 4-hydroxytamoxifen (4OHT)-regulated systems, have been shown to function in cultured cells.^51^ In addition, in principle, electroporation-based mRNA delivery could be further simplified by co-injecting recombinant PBase protein together with donor DNA directly into the pronucleus, which would eliminate the need for mRNA delivery and could enable immediate transposase activity at the time of donor entry. Although recombinant PBase has been reported,^52^ it is not currently commercially available to our knowledge.

We examined allelic integration bias using crosses between PWK sperm and B6J oocytes. The primary objective of this experiment was not simply to determine whether transgene integration preferentially occurs in one pronucleus over the other, but rather to assess whether integration is completed before or after PN fusion, a key determinant of potential F_0_ mosaicism. If donor DNA injected into the male pronucleus were fully integrated prior to PN fusion with no subsequent integration events, insertions would be expected to be restricted to the paternal allele; however, integrations were also detected on the maternal allele, indicating that donor DNA integration can continue beyond PN fusion, consistent with the possibility of ongoing mosaic integration.

Single-cell BC tracing further resolved the timing and cell-type distribution of integration events: In F_0_ embryos, we consistently observed two major BC-defined clusters spanning multiple tissues—consistent with predominant integration at the two-cell stage—while ∼14% of BCs detected in genomic DNA were not captured as transcripts, indicating post-integration silencing/position effects in a subset of sites. Together, these findings indicate that early *piggyBac* integration can support F_0_-based phenotypic assessment, while also highlighting residual, quantifiable sources of heterogeneity that should be considered when interpreting F_0_ phenotypes. While F_0_ mosaicism has traditionally been assessed primarily through germline transmission when the goal is stable line establishment, F_0_-based phenotypic analysis requires evaluation of transgene distribution within the F_0_ animal itself. In this context, transgene presence and copy-number distribution should be relatively uniform across cells within each tissue, because substantial cell-to-cell variability, particularly in the context of ScKiP, could create mixtures of edited and non-edited cells that confound phenotypic interpretation. Because there is no established approach to quantitatively evaluate within-animal cellular distribution of transgenes at single-cell resolution, the combination of DNA barcoding and scRNA-seq was essential for assessing cellular uniformity relevant to F_0_-based analysis.

Although our primary focus was on mice, improving *piggyBac* transgenesis has implications for other species. In rat, *piggyBac* plasmid injection typically yields Tg efficiency below 30%,^47^^,^^53^^,^^54^ whereas one study achieved 33%–100% (mean ∼80%) using mPBase mRNA PN microinjection.^55^ Introducing mPBase mRNA earlier, as we did in this study, may further improve rat transgenesis. For livestock, reports of *piggyBac* in zygotes are scarce,^56^^,^^57^ with somatic cell nuclear transfer being the standard, technically demanding approach, with a low success rate. While no reports have described the testing of mPBase mRNA in livestock, to the best of our knowledge, our findings suggest that this is a promising avenue.

Although *in vitro* studies using cultured cells provide a useful and rapid approach for evaluating tissue-specific gene functions, they cannot fully recapitulate the complex cellular interactions, tissue architecture, and physiological environment present *in vivo*. Tissue-specific cKO mice are therefore invaluable for elucidating gene functions *in vivo,* but their generation is time-consuming because it requires the production of floxed and Cre-Tg strains and the performance of multiple crosses. Recently, several rapid alternatives have been developed. One approach introduces CRISPR-Cas9 ribonucleoproteins into mice via nanoparticles, enabling efficient KO in tissues such as liver, muscle, brain, kidney, and lung.^18^^,^^19^^,^^20^^,^^21^ Another involves introducing a floxed allele into zygotes derived from tissue-specific Cre-Tg mice using KI technology.^22^ A third uses adeno-associated virus (AAV) to deliver gRNAs into mice expressing Cas9 in specific tissue, and a fourth combines AAV-mediated gRNA delivery with a tissue-specific Cre expression vector in mice harboring a Cre-loxP-dependent Cas9 cassette. Although powerful, these methods differ in targetable tissues, efficiency, and technical complexity. Careful selection depending on the purpose of the research is therefore required. In this study, we developed the ScKiP method as another rapid option for cKO experiments and tested its utility. For rigorous evaluation, we targeted *Prmt1* and *Kit*, whose systemic KO is embryonically lethal, requiring cKO approaches to study postnatal functions. Although ScKiP was not strictly tissue-specific, likely due to positional effects, it yielded the expected phenotype. Notably, KO cells can be readily identified by the loss of EGFP fluorescence, a feature unique to ScKiP. Thus, ScKiP provides a practical strategy for rapid screening of gene functions *in vivo.* In mice, where many validated tissue-specific Cre lines are available, combining such Cre lines with *piggyBac*-mediated delivery of floxed Cas9 and gRNAs may represent a practical variant of the ScKiP concept.

Given the inherent variability associated with F_0_-based genome editing, ScKiP is positioned not as a replacement for conventional Cre-loxP-based cKO strategies, but as a complementary, high-throughput *in vivo* screening platform designed to rapidly narrow down candidate genes based on early phenotypic outcomes. Conventional cKO models remain essential for hypothesis-driven studies requiring precise control of allele structure and tissue specificity, whereas ScKiP provides rapid access to *in vivo* phenotypes that can guide prioritization. Accordingly, these factors should be considered when interpreting F_0_ phenotypes, and key findings should be confirmed by downstream validation using rigorously controlled genetic models. The limitation and practical considerations inherent to ScKiP and other F_0_-based approaches are discussed in detail in the limitations of the study section.

In summary, our optimized *piggyBac* transgenesis method enables highly efficient Tg production, including with large DNA constructs, and supports rapid F_0_ phenotyping through ScKiP. These advances should not only accelerate functional genomics but also contribute to animal welfare by reducing the number of animals used and streamlining experimental workflows.

### Limitations of the study

Despite the high efficiency of the optimized *piggyBac* platform and its utility for F_0_-based analysis, several limitations should be considered.

First, although single-cell BC tracing provides a high-resolution view of integration timing and tissue distribution, rare late integration events or transcriptionally silent insertions may be underrepresented due to technical dropout and/or post-integration silencing. Accordingly, the observed profiles should be interpreted as quantitative estimates rather than exhaustive enumeration of all integration events.

Second, allelic integration bias was assessed using PWK sperm and B6J oocytes, which enabled discrimination of paternal and maternal alleles. However, because sperm and oocytes were derived from different genetic backgrounds, strain-dependent influences on allelic outcomes cannot be fully excluded. Reciprocal crossing experiments would ideally address this point. However, oocyte recovery from wild-derived PWK females is inefficient, and their husbandry and breeding are technically demanding, thereby limiting the feasibility of obtaining sufficient sample numbers.^58^ Therefore, we interpret allelic patterns primarily in the context of integration timing and mosaicism, while acknowledging this experimental constraint.

Third, while ScKiP enables rapid conditional gene disruption in F_0_ animals, tissue specificity was not complete, likely reflecting positional effects and/or ectopic Cre activity associated with random transposon integration. Such ectopic Cre activity may lead to constitutive Cas9 expression and genome editing outside the intended target tissue, thereby confounding interpretation of ScKiP-derived phenotypes. As shown in Figure 5A, positional effects can be partially mitigated by incorporating insulator elements. Moreover, because *piggyBac* Tg approach often results in multiple transgene copies integrated at distinct genomic loci within the same individual, positional effects may be averaged across the genome, potentially stabilizing phenotypic outcomes compared with single-copy random integration. Nevertheless, residual integration site-dependent variability may remain, and tissue specificity can vary among F_0_ animals. In addition, ScKiP relies on CRISPR-Cas9-mediated editing downstream of Cre activation. Therefore, KO outcomes are inherently subject to mosaicism and variability across cells and F_0_ animals. Editing events may yield biallelic, monoallelic, or no disruption and a fraction of indels may be in-frame, potentially generating hypomorphic or altered-function alleles rather than complete loss-of-function. Moreover, Cre activity monitored by a fluorescent reporter does not guarantee uniform Cas9-mediated loss-of-function editing across all target cells, reflecting the fundamental difference in efficiency between Cre-mediated recombination and Cas9-mediated genome editing. The Prmt1 experiment illustrates that a phenotype can be detected even when bulk-tissue editing levels are modest; however, this should not be generalized to all targets, because some genes or tissues may require substantially higher levels of disruption to reveal a phenotype. Therefore, the absence of an overt phenotype in F_0_ animals should not be interpreted as evidence that tissue-specific disruption of the target gene has no biological effect, unless sufficient editing efficiency, cellular coverage, and tissue specificity have been confirmed. To mitigate these technical limitations, gRNA design can be informed by *in silico* prediction tools^59^^,^^60^^,^^61^ to prioritize guides with a higher likelihood of generating loss-of-function alleles and to target exons, splice junctions, or termination codons shared across isoforms. In addition, taking advantage of the large cargo capacity of *piggyBac*, multiple gRNA expression cassettes can be incorporated simultaneously to reduce reliance on a single cleavage event and increase the robustness of gene disruption. Potential toxicity associated with constitutive Cas9 expression also remains an important concern. In our cardiac cKO experiments (Figures 5A–5G), despite the expectation that most offspring would be Tg under the optimized conditions, only a single Tg animal was ultimately obtained, raising the possibility that Cas9 expression adversely affected cardiac development and contributed to embryonic lethality. This low recovery also indicates that ScKiP may not always yield sufficient F_0_ numbers for robust phenotypic analysis in a single experimental round, particularly when ectopic Cre/Cas9 activity or target-gene disruption affects embryonic or postnatal viability. Off-target or non-specific phenotypes, as well as reduced recovery of viable animals due to Cas9 expression, therefore remain important challenges to be addressed. Consequently, ScKiP should be interpreted as a rapid *in vivo* screening approach to prioritize candidate genes, with key findings requiring downstream validation using rigorously controlled genetic models, including germline-transmitted lines where appropriate. Nevertheless, even when these risks are taken into account, the ability to obtain early *in vivo* phenotypic information within weeks, before committing to conventional cKO line generation that typically requires months to years, provides a practical advantage for prioritizing candidates.

Fourth, optimization of transposase dosage and transgene copy number should be considered in a purpose-dependent manner. Increasing integration events may improve robustness of transgene expression for F_0_ screening, but it also carries inherent risks, including disruption of endogenous genes and cellular toxicity due to excessive expression of exogenous genes. Accordingly, lower-copy integration conditions may be preferable for applications focused on stable line establishment and germline transmission, whereas higher-copy conditions may be advantageous when the goal is robust phenotypic screening in F_0_ animals. In this context, reducing the concentration of PBase mRNA and/or employing a lower-activity transposon system including piggyBat may represent useful options depending on experimental goals.^62^ In addition, residual F_0_ mosaicism should be considered when interpreting F_0_ phenotypes. Depending on the experimental goal, establishing germline-transmitted lines from efficiently generated F_0_ animals may ultimately yield clearer results, and may in some cases be more efficient and require fewer animals than direct F_0_ analysis.

Finally, the present study focused primarily on mice and a limited number of representative genes, and therefore broader validation across diverse loci, tissues, and experimental contexts will be required to establish general applicability. In addition, although the optimized *piggyBac* strategy may be informative for other species, its utility beyond mice remains to be tested experimentally.

## Resource availability

### Lead contact

Requests for further information and resources should be directed to and will be fulfilled by the lead contact, Masatsugu Ema (mema@belle.shiga-med.ac.jp).

### Materials availability

All unique reagents and plasmids generated in this study are available from the lead contact upon reasonable request. Distribution may require a completed materials transfer agreement (MTA).

### Data and code availability

The sequencing data generated in this study have been deposited in Gene Expression Omnibus under accession numbers GEO: GSE306203, GSE308034, and GSE309826 and are publicly available. No original code was generated in this study. Any additional information required to reanalyze the data reported in this paper is available from the lead contact upon request.

## Acknowledgments

pcDNA3.1-EGFP-poly(A) plasmid was kindly provided by Dr. Kazuo Yamagata (Kindai University). We thank the Wellcome Sanger Institute for kindly providing the hyPBase cDNA. This work was supported by 10.13039/501100001691JSPS KAKENHI grant numbers 21K05988 and 23H03860 (to E.O.), 20K22611 (to S. Matsumoto), and 21H02388 (to M.E.) and JST FOREST Program, grant number JPMJFR221H (to S. Mizuno). We thank K. Shiina for NGS analysis and staff at Central Research Laboratory at Shiga University of Medical Science and Single-cell Genome Information Analysis Core (SignAC) at WPI-ASHBi, Kyoto University, for their support. This research was partially supported by Research Support Project for Life Science and Drug Discovery (Basis for Supporting Innovative Drug Discovery and Life Science Research [BINDS]) from 10.13039/100009619AMED under grant number JP25ama121016. We also thank Edanz (https://jp.edanz.com/ac) for editing a draft of this manuscript. The graphical abstract, Figure 3 and S4 were created in BioRender. Okamura, E. (2026) https://BioRender.com/sfv20u4; https://BioRender.com/6lepdkn.

## Author contributions

Conceptualization, E.O., S. Matsumoto, E.M., S. Mizuno, and M.E.; methodology, E.O., S. Matsumoto, E.M., N. Masuyama, Y.K., H.M., S. Mizuno, and M.E.; investigation, E.O., S. Matsumoto, E.M., K. Murata, Y.T., T.T.H.D., H.S., A.K., W.K., N. Mikami, T.E., K. Morimoto, K.K., T. Matsumoto, N. Masuyama, Y.K., M.M., T. Morimura, H.M., and S. Mizuno; formal analysis, E.O., S. Matsumoto, E.M., K. Murata, S. Mizuno, G.N., K.W., N.Y., and M.E.; data curation, E.O., S. Matsumoto, E.M., A.K., N. Masuyama, Y.K., H.M., S. Mizuno, G.N., K.W., N.Y., and M.E.; software, A.K., N. Masuyama, and Y.K.; visualization, E.O., S. Matsumoto, E.M., K. Murata, N. Masuyama, Y.K., H.M., S. Mizuno, and M.E.; resources, F.S., S.T., H.A., K.W., N.Y., S. Mizuno, and M.E.; funding acquisition, E.O., S. Matsumoto, E.M., H.A., N.Y., S. Mizuno, and M.E.; supervision, N.Y., S. Mizuno, and M.E.; project administration, E.O., S. Matsumoto, E.M., S. Mizuno, and M.E.; writing – original draft, E.O., S. Matsumoto, E.M., S. Mizuno, and M.E.; writing – review and editing, all authors.

## Declaration of interests

The authors declare no competing interests.

## Declaration of generative AI and AI-assisted technologies in the writing process

During the preparation of this work, the authors used ChatGPT to improve the clarity, readability, and grammatical accuracy of the manuscript text. After using this tool, the authors reviewed and edited the content as needed and took full responsibility for the content of the published article.

## STAR★Methods

### Key resources table

**Table:** 

REAGENT or RESOURCE	SOURCE	IDENTIFIER
**Antibodies**		

Anti-Cas9 Monoclonal Antibody	Nippon Gene	Cat# 310-08431
Polyclonal anti-GFRα1 (Goat)	R&D Systems	Cat# AF560; RRID: AB_2110307

**Bacterial and virus strains**		

NEB® Stable Competent *E. coli* (High Efficiency)	NEB	Cat#C3040I

**Chemicals, peptides, and recombinant proteins**		

CARD HyperOva (Superovulation Reagent for mouse)	Kyudo	Cat#KYD-010-06-EX
CARD FERTIUP Preincubation Medium	Kyudo	Cat#KYD-002-05-EX
CARD Medium	Kyudo	Cat#KYD-003-EX
KSOM medium	Ark Resource	N/A
Opti-MEM™ I Reduced Serum Medium	Thermo Fisher Scientific	Cat#31985-062
M2 Medium	Sigma-Aldrich	Cat#M7167
equine chorionic gonadotropin	ASKA Animal Health	N/A
human chorionic gonadotropin	ASKA Animal Health	N/A
Phenol:Chloroform:Isoamyl Alcohol 25:24:1 Mixed, pH 7.9	Nacalai Tesque	Cat#25970-56
Hae III (BsuR I)	Thermo Fisher Scientific	Cat#FD0154
EcoRI-HF	New England Biolabs	Cat#R3101L
DNA Ligation Kit <Mighty Mix>	Takara	Cat#6023
Agar Powder	Nacalai Tesque	Cat#01028-85
Agarose for ≥1kbp fragment (Fine Powder)	Nacalai Tesque	Cat#02468-66
Amersham Hybond^TM^ -N^+^	GE Healthcare	Cat#RPN203B
Whatman® 3 MM CHR CHROMATOGRAPHY PAPER	cytiva	Cat#3030-909
DIG Easy Hyb	Roche Diagnostics	Cat#11603558001
Blocking Reagent	Roche Diagnostics	Cat#11096176001
Anti-Digoxigenin-AP Fab fragments	Roche Diagnostics	Cat#11093274910
CDP-Star	Roche Diagnostics	Cat#11685627001
DIG DNA Labeling Mix, 10× Conc.	Roche Diagnostics	Cat#11277065910
Mitomycin C	Sigma-Aldrich	Cat#M0503-2 MG
DMEM (High Glucose)	Nacalai Tesque	Cat#08458-45
KOD One™ PCR Master Mix –Blue–	Takara	Cat#KMM-201
KnockOut™ SR	Thermo Fisher Scientific	Cat#10828-028
StemSure® 2-Mercaptoethanol	Wako	Cat#198-15781
Sodium Pyruvate	Thermo Fisher Scientific	Cat#11360-070
Penicillin Streptomycin	Thermo Fisher Scientific	Cat#15140-122
CultureSure® CHIR99021	Wako	Cat#034-23103
PD0325901	Wako	Cat#162-25291
mouse LIF	In-house	N/A
0.25% Trypsin-EDTA	Thermo Fisher Scientific	Cat#25200-072
PrimeSTAR® MAX DNA Polymerase	Takara	Cat#R045A

**Critical commercial assays**		

mMESSAGE mMACHINE™ T7 ULTRA Transcription Kit	Thermo Fisher Scientific	Cat#AM1345
mMESSAGE mMACHINE™ T7 Transcription Kit	Thermo Fisher Scientific	Cat#AM1344
MEGAclear™ Transcription Clean-Up Kit	Thermo Fisher Scientific	Cat#AM1908
ddPCR Supermix for Probes (No dUTP)	Bio-Rad	Cat#1863023
TaqMan™ Copy Number Reference Assay, mouse, Tfrc	Thermo Fisher Scientific	Cat#4458366
FastGene Gel/PCR Extraction Kit	Nippon Genetics	Cat#FG-91302
Monarch® DNA Gel Extraction Kit	New England BioLabs	Cat#T1020
iSeq 100 i1 Reagent v2 (300-cycle)	Illumina	Cat#20031371
3′ CellPlex Kit Set A, 48 rxns	10× Genomics	Cat#PN-1000261
3′ Feature Barcode Kit, 16 rxns	10× Genomics	Cat#PN-1000262
Chromium NextGEM Single Cell 3′ Kit v3.1, 16 rxns	10× Genomics	Cat#PN-1000268
Dual Index Kit TT Set A, 96 rxns	10× Genomics	Cat#PN-1000215
Chromium Next GEM Chip G Single Cell Kit, 48 rxns	10× Genomics	Cat#PN-1000120
RED/ET recombination technique	Gene Bridges	N/A

**Deposited data**		

H3K4me3 ChIP-seq for zygotes and early 2-cell embryos from PWK/PhJ (male) x C57BL/6N (female) crosses	Zhang et al.^28^	GEO: GSE71434
scRNA-seq data of E11.5 mouse embryo with DNA barcode-linked GFP	This paper	GEO: GSE306203, GSE309826
Amplicon-seq data of mouse ES cells and E11.5 mouse embryo with DNA barcode-linked GFP	This paper	GEO: GSE308034

**Experimental models: Cell lines**		

Mouse: mouse ES cells *Tg(CAG-EGFP-BC)*	This paper	N/A

**Experimental models: Organisms/strains**		

Mouse: C57BL/6J	Charles River Laboratories	N/A
Mouse: C57BL/6N	Japan SLC	N/A
Mouse: Crl:CD1(ICR)	Charles River Laboratories	N/A
Mouse: PWK/PhJ	RIKEN BioResource Center	RBRC00213
Mouse: BDF1	Japan SLC	N/A
Mouse: C57BL/6N-Gt(ROSA)26Sor<tm1(CAG-EGFP/dsRed)Utr>/Rbrc	Hasegawa et al.^35^ RIKEN BioResource Center	RBRC04874
Mouse: CdEC flox mouse: C57BL/6J-Gt(ROSA)26Sor<em1(floxed-EGFP-Cas9NmC)Utr>	This paper, RIKEN BioResource Center	RBRC12114

**Oligonucleotides**		

Primers to generate ddPCR and Southern Blotting probes	See Table S7	N/A
PCR primers	See Table S7	N/A
Primers for barcode-specific PCR	See Table S7	N/A
Primers for transgene-specific library construction	See Table S7	N/A
Primers for producing DNA barcode fragment	See Table S7	N/A

**Recombinant DNA**		

Plasmid: pcDNA3.1-EGFP-poly(A)	Yamagata et al.^63^	N/A
Plasmid: ITR-CAG-EGFP-BC-pA-ITR (pNM1419)	This paper	N/A
Plasmid: ITR-CAG-EGFP-pA-ITR	This paper	N/A
Plasmid: ITR-cHS4-UbC-EGFP-2A-mCherry-pA-cHS4-ITR	This paper	N/A
Plasmid: ITR-cHS4-Myh6-NLS-EGFP-pA-(CAG-tdTomato-pA)-cHS4-ITR	This paper	N/A
Plasmid: ITR-CAG-BFP-pA-ITR	This paper	N/A
Plasmid: ITR-CAG-EGFP-pA-ITR	This paper	N/A
Plasmid: ITR-CAG-tdTomatoP-pA-ITR	This paper	N/A
Plasmid: ITR-CAG-E2Crimson-pA-ITR	This paper	N/A
Plasmid: BAC clones covering *Flk1* locus (ITR-cHS4-CAG-KO-pA-cHS4-(Flk1-EGFP-pA))	Invitrogen	RP22-125B24
Plasmid: ITR-cHS4-Myh6-Cre-pA-U6-Prmt1 gRNA-cHS4-ITR	This paper	N/A
Plasmid: ITR-U6-Kit gRNA-Ddx4-CreERT2-pA-ITR	This paper	N/A
Plasmid: CAG-PBase	Kim et al.^64^	N/A

**Software and algorithms**		

Ubuntu (v20.04)	Canonical Ltd.	https://jp.ubuntu.com/
Cell Ranger (v7.2.0)	10× Genomics	https://www.10xgenomics.com/support/jp/software/cell-ranger/latest
R (v4.4.1)	R Foundation for Statistical Computing	https://www.r-project.org/
Seurat (v5.1.0)	Hao et al.^65^	https://github.com/satijalab/seurat
SoupX (v1.6.2)	Young et al.^66^	https://github.com/constantAmateur/SoupX
DoubletFinder (v2.0.4)	McGinnis et al.^67^	https://github.com/chris-mcginnis-ucsf/DoubletFinder
RECODE (v1.0.0)	Imoto et al.^68^	https://github.com/yusuke-imoto-lab/RECODE
dplyr (v1.1.4)	Wickham et al.^69^	https://github.com/tidyverse/dplyr
ggplot2 (v3.5.1)	Wickham.^70^	https://github.com/tidyverse/ggplot2
clusterProfiler (4.12.0)	Wu et al.^71^	https://github.com/YuLab-SMU/clusterProfiler?tab=readme-ov-file
escape (v2.0.0)	Borcherding et al.^72^	https://github.com/BorchLab/escape
starcode (v1.4)	Zorita et al.^73^	https://github.com/gui11aume/starcode
ComplexHeatmap (v2.20.0)	Gu et al.^74^	https://bioconductor.org/packages/release/bioc/html/ComplexHeatmap.html
bowtie2	Langmead and Salzberg^75^	https://github.com/BenLangmead/bowtie2
MACS2	Zhang et al.^76^	https://github.com/hbctraining/Intro-to-ChIPseq
deepTools	Ramírez et al.^77^	https://deeptools.readthedocs.io/en/latest/
IGV	Robinson et al.^78^	https://igv.org/
Vevo LAB software	FUJIFILM	https://www.visualsonics.com/faq/answer/downloading-vevo-lab

### Experimental model and study participant details

#### Mice

All mice used in this study were Mus musculus. C57BL/6J and ICR mice were purchased from Charles River Laboratories (Yokohama, Japan). C57BL/6N and BDF1 mice were purchased from Japan SLC. PWK/PhJ mice (RBRC00213) were obtained from RIKEN BioResource Research Center. C57BL/6N-Gt(ROSA)26Sor<tm1(CAG-EGFP/dsRed)Utr>/Rbrc mice (RBRC04874) and C57BL/6J-Gt(ROSA)26Sor<em1(floxed-EGFP-Cas9NmC)Utr> mice (RBRC12114) were used as genetically modified mouse lines as indicated in the relevant experiments.

Mice were maintained in plastic cages under pathogen-free conditions in a room maintained at 23.5 ± 2.5°C and 52.5 ± 12.5% relative humidity under a 14 h light:10 h dark cycle, with *ad libitum* access to food and water. Fertilized eggs, preimplantation embryos, E9.5 and E11.5 embryos, neonatal mice, juvenile mice, and adult mice were used depending on the experiment. For *in vitro* fertilization experiments, oocytes were collected from 10 to 14-week-old C57BL/6J females and sperm were collected from 12 to 16-week-old C57BL/6J males. For ICSI-Tr experiments, MII oocytes were collected from BDF1 females and sperm were collected from adult C57BL/6N males. ICR females were used as pseudopregnant recipients.

Both male and female embryos or offspring were included unless otherwise specified. Sex was not determined for preimplantation and fetal-stage transgenesis experiments because the primary outcomes were transgene presence, fluorescence, copy number, integration pattern, and barcode distribution at embryonic stages. For postnatal phenotypic analyses, sex and age/developmental stage are described in the relevant method sections.

All animal experiments were carried out in accordance with the Fundamental Guidelines for Proper Conduct of Animal Experiments and Related Activities in Academic Research Institutions under the jurisdiction of the Ministry of Education, Culture, Sports, Science and Technology, and Guidelines for Proper Conduct of Animal Experiments from the Science Council of Japan. All animal experimental procedures were approved by the Animal Care and Use Committee of Shiga University of Medical Science (2020-6-21) and the Institutional Animal Experiment Committee of the University of Tsukuba (22-019, 21–155).

#### Mouse embryonic stem cells

Mouse embryonic stem cell lines carrying DNA barcode-linked GFP transgene cassettes were established from inner cell masses of mouse blastocysts generated in this study. Cells were cultured at 37°C in a humidified incubator with 5% CO2 on mitomycin C-treated mouse embryonic fibroblasts in 2iL medium, as described below. The sex of the established mES cell lines was not determined. Cell line authentication was not performed beyond genotyping, fluorescence confirmation, and barcode validation described in this study.

The cell lines were not independently authenticated by STR profiling or other genetic authentication methods in this study. The cells were not tested for mycoplasma contamination.

### Method details

#### *In vitro* transcription

The template plasmid for *in vitro* transcription of piggyBac transposase mRNA, either mPBase^25^ or hyPBase,^26^ was generated by replacing the EGFP sequence in pcDNA3.1-EGFP-poly(A)^63^ with the mPBase or hyPBase cDNA sequence; the hyPBase cDNA was kindly provided by the Wellcome Sanger Institute. After linearization of this template plasmid with the XhoI restriction enzyme, mPBase or hyPBase mRNA was synthesized using the mMESSAGE mMACHINE T7 ULTRA Transcription Kit (AM1345; Thermo Fisher Scientific, Waltham, MA, USA) or the mMESSAGE mMACHINE T7 Transcription Kit (AM1344; Thermo Fisher Scientific). The synthesized RNA was purified using the MEGAclear Transcription Clean-Up Kit (#AM1908; Thermo Fisher Scientific).

#### Construction and validation of the DNA barcode-linked GFP donor library

The DNA barcode (BC)-linked GFP donor plasmid (pNM1419) was constructed from a piggyBac transposon donor backbone in which an EGFP expression cassette driven by the CAG promoter was flanked by the 5′ and 3′ piggyBac ITRs. The 20bp BC-containing fragment was amplified by PCR using PrimeSTAR Max DNA Polymerase (Takara Bio, Kusatsu, Japan) with primers BC-fragment-F and -R, followed by agar gel verification and colum purification. The donor backbone and PCR-derived insert were digested with AgeI and PpuMI (New England BioLabs, Ipswich, MA, USA) and purified, and the BC fragment was inserted by T4 DNA ligase-mediated ligation. The ligation products were purified and transformed into NEB Stable competent cells (New England BioLabs) to generate the plasmid pool.

To generate a high-complexity donor library, plasmids were pooled from approximately 15,000 independent E. coli colonies. To empirically assess library diversity, the pooled plasmid library was re-transformed into E. coli, and 98 colonies were randomly selected for barcode sequencing (Table S6). Among these, 96 unique barcode sequences were identified, with only a single duplicated pair observed. Based on a standard birthday-problem approximation, the expected number of duplicated barcode pairs at this sampling depth is approximately 0.32 consistent with the observed low duplication rate and supporting the interpretation that the library retained the intended complexity. Given that the maximum number of integrated transgene copies per animal in this study was approximately 25 (Figure 1F), the probability of barcode collision within an individual is expected to be negligible for downstream single-cell-level analysis of transgene distribution.

#### *In vitro* fertilization, electroporation, and microinjection

Fertilized eggs were obtained by *in vitro* fertilization using a method described elsewhere.^79^ Briefly, oocytes were collected from oviducts of 10–14-week-old C57BL/6J females superovulated by the intraperitoneal administration of CARD HyperOva (Kyudo, Tosu, Japan), followed by human chorionic gonadotropin (hCG). Sperm were collected from the caudal epididymis of 12–16-week-old C57BL/6J males, and then preincubated in Fertiup Mouse Sperm Preincubation Medium (Kyudo). Insemination was performed in CARD medium (Kyudo), followed by incubation at 37 °C in an atmosphere containing 5% CO2 for 3 to 6 h. Fertilized eggs were washed to remove cumulus cells and sperm, and incubated in Potassium Simplex Optimized Medium (KSOM) (Ark Resource, Kumamoto, Japan) until electroporation. Electroporation of fertilized eggs was performed in Opti-MEM I Reduced Serum Medium (Thermo Fisher Scientific) containing piggyBac mRNA using Super Electroporator NEPA21 (Nepa Gene, Ichikawa, Japan), as described elsewhere^80^ with minor modifications. In this study, the poring pulse was set as follows: 225 V, 2 ms pulse width, 50 ms pulse interval, 4 pulses, 10% attenuation rate, and + polarity. Meanwhile, the transfer pulse was set as follows: 20 V, 50 ms pulse width, 50 ms pulse interval, 5 pulses, 40% attenuation rate, and ± polarity. The eggs were incubated until pronuclei were clearly visible. The transposon DNA was microinjected under a microscope equipped with a micromanipulator and Femtojet (Eppendorf, Hamburg, Germany). The injected one-cell embryos were cultured in KSOM until the two-cell stage and then transferred into pseudopregnant ICR mice. For some experiments, following electroporation, eggs were transferred to M2 medium (Sigma-Aldrich, St. Louis, MO, USA) and kept overnight at 4°C. The next morning, microinjection was performed and the fertilized eggs were subsequently transferred into surrogate mothers at the one-cell stage.

#### Modified ICSI-Tr with mPBase mRNA injection

Intracytoplasmic sperm injection (ICSI) was performed as previously described^43^ with minor modifications. Briefly, MII oocytes were collected from superovulated BDF1 (Japan SLC) females after administration of equine chorionic gonadotropin (eCG; ASKA Animal Health, Tokyo, Japan) and human chorionic gonadotropin (hCG; ASKA Animal Health, Tokyo, Japan). mPBase mRNA was injected into the oocyte cytoplasm using piezo-driven micromanipulators, followed by SrCl2 treatment, when required, prior to ICSI. Sperm were collected from the cauda epididymides of C57BL/6N (Japan SLC) adult males. Freezing of sperm was conducted at −30°C, when necessary. Sperm tails were removed with a piezoelectric pulse and only the sperm heads were injected into oocytes with donor DNA solution. After ICSI, the zygotes were cultured *in vitro* and two-cell-stage embryos were transplanted into the oviduct of surrogate ICR (Japan SLC) females.

#### Genotyping of the embryo

Embryos at embryonic day 11.5 (E11.5) were collected from the uteri of surrogate mothers after euthanasia and the placenta and yolk sac were removed. The fluorescent signal was captured under a fluorescent stereoscopic microscope (FL5; Leica Microsystems, Wetzlar, Germany) equipped with a camera (DP73; Olympus, Tokyo, Japan). Genomic DNA from the embryos was obtained by phenol/chloroform extraction. Genotyping PCR was performed using a primer set to detect the GFP sequence using conventional PCR (T100; Bio-Rad, Hercules, CA, USA) (Table S7). To determine the copy number of the transgene, droplet digital PCR (ddPCR) was performed using ddPCR Supermix for Probes (No dUTP) (Bio-Rad) with the Qx200 Droplet Digital PCR system (Bio-Rad) and the GFP and Tfrc Primer/Probe Set (Table S7), and Hae III restriction enzyme (Thermo Fisher Scientific).

#### Determination of Tg integration sites by inverse PCR

Inverse PCR was performed using a method described elsewhere^81^ with minor modifications. In this study, 3 μg of DNA was digested by EcoRI and the digested DNA was then self-ligated using DNA Ligation Kit (Mighty Mix; Takara Bio) after purification by ethanol precipitation. Junction regions were amplified using KOD One PCR Master Mix -Blue- (TOYOBO, Osaka, Japan) through a three-round PCR workflow. First, outward-facing transgene-specific primers were used to amplify transposon-genome junction fragments. A 2nd-round PCR using nested transgene-specific primers was then performed to enrich junction products and reduce nonspecific amplification. For both the 1st- and 2nd-round PCR, thermal cycling conditions were as follows: 98°C for 2 min; 25 cycles of 98°C for 10 s and 68°C for 1 min; with a final extension at 68°C for 5 min. Finally, the 3rd -round PCR was performed using DNA barcode-specific primers to assign each amplicon to an individual transgene. The barcode-specific primer sets were designed for each detected barcode sequence based on the barcode calls obtained by Amplicon-seq (see “Amplicon-seq for integrated transgene number detection” below). The thermal cycling conditions for each barcode-specific PCR were as follows: 98°C for 2 min; 35 cycles of 98°C for 10 s, 60°C for 5 s and 68°C for 1 min; with a final extension at 68°C for 5 min. After electrophoresis, the amplicons were extracted and purified using Monarch DNA Gel Extraction Kit (New England BioLabs). Sanger sequencing was outsourced to Eurofins Genomics (Tokyo, Japan). Integrated loci were identified using the BLAT function in the UCSC Genome Browser (https://genome.ucsc.edu/). The primer sequences used for inverse PCR are shown in Table S7. Because EcoRI is a 6-bp cutter, some junctions may be recovered less efficiently if restriction sites are distant. However, integration counts showed good concordance with Southern blotting and ddPCR (Figure 2B).

#### Southern blotting

Genomic DNA was extracted from mouse embryos with phenol and chloroform and then purified by ethanol precipitation. After digestion of 5 μg of genomic DNA by EcoRI, the concentration was measured using a Quantus Fluorometer (Promega, Madison, WI, USA). One microgram of digested DNA was electrophoresed in 1% agarose gel. The DNA was then transferred onto a nylon membrane (Amersham Hybond-N+; GE Healthcare, Chicago, IL, USA) using Whatman 3 MM CHR chromatography paper (Cytiva, Tokyo, Japan), and the membrane was UV-crosslinked for 2 min. Heat-denatured salmon sperm DNA at 66.6 μg/mL was added to the hybridization buffer to reduce nonspecific hybridization. 50 ng/mL of heat-denatured DIG-labeled probe was hybridized in DIG Easy Hyb (Roche Diagnostics, Mannheim, Germany) overnight at 52°C. GFP and Gusb probes were synthesized using PCR DIG Probe Synthesis Kit (Roche Diagnostics). After hybridization and washing, the membrane was blocked using Blocking Reagent (Roche Diagnostics) before incubation with anti-digoxigenin-AP Fab fragments (Roche). The bands were detected by enhanced chemiluminescence using CDP-Star (Roche Diagnostics) and ImageQuant LAS 4000 Mini (GE Healthcare). All probes were synthesized by PCR using DIG DNA Labeling Mix, 10× Conc. (Roche Diagnostics). The primer sequences used to generate the probes for Southern blotting are shown in Table S7.

#### Analysis of ChIP-seq data

H3K4me3 ChIP-seq data for zygote and early two-cell-stage mouse embryo from a cross between PWK/PhJ (male) and C57BL/6N (female) were downloaded from the Gene Expression Omnibus (GEO) database (GEO: GSE71434).^28^ The reads were aligned to the mouse reference genome (mm9) using bowtie2 v2.3.5.1.75 The peaks were called by MACS2 v2.2.7.176 with the option “-p 0.01.” Genome coverage tracks were generated using the bamCoverage function in deepTools v3.5.177 with the parameters “--normalizeUsing RPGC --binSize 1.” The peaks of H3K4me3 were visualized using Integrative Genomics Viewer (IGV) software.^78^

#### Establishment and culture of mouse embryonic stem cell lines with DNA barcode

Inner cell mass (ICM) was isolated from mouse blastocyst with DNA barcode-linked GFP by immunosurgery. For the establishment of embryonic stem (ES) cell lines, ICM was cultured on mitomycin C-treated mouse embryonic fibroblasts (MEFs) in Dulbecco’s Modified Eagle’s Medium (DMEM, high glucose; Nacalai Tesque, Kyoto, Japan) supplemented with 20% KnockOut Serum Replacement (KSR; Thermo Fisher Scientific), 1% non-essential amino acids (NEAAs; Thermo Fisher Scientific), 1 mM sodium pyruvate (Thermo Fisher Scientific), 0.1 mM 2-mercaptoethanol (Wako, Osaka, Japan), 1% penicillin-streptomycin (Thermo Fisher Scientific), 1000 U/ml mouse LIF (made in-house), 1 μM PD0325901 (Wako), and 3 μM CHIR99021 (Wako) (2iL). A single colony was manually picked up and cultured on a 0.1% gelatin-coated plate under 2iL conditions.

#### Amplicon-seq for integrated transgene number detection

Extracted and purified genomic DNA from mES BC lines and E11.5 mouse Tg embryos was amplified using transgene-specific primers with Illumina index sequences (Table S7; Figure S4C). PCR was performed using KOD One PCR Master Mix -Blue- (TOYOBO). The amplicons were purified using the FastGene Gel/PCR Extraction Kit (Nippon Genetics, Tokyo, Japan). The Amplicon-seq libraries were sequenced on the iSeq 100 (Illumina) sequencing platform using 300 bp single-end sequencing from both 5′ and 3′ ends of the libraries.

From the fastq files, the regions of the BC sequence were extracted using reference guide sequences, which were 10 bp upstream and downstream of the DNA barcode. Similar BC sequences, which might be caused by sequencing error, were summarized by starcode.^73^ Because abnormal BC sequences that were probably caused by sequencing error were detected by NGS, we developed an algorithm to determine the optimal threshold for defining such errors. To detect bona fide BC sequences, the fold change of the reads was calculated between each BC sequence using the read depth matrix ordered from top to bottom rank, with the highest value being used as a threshold. The raw data are available in the GEO database (GSE308034).

#### Single-cell RNA-seq library construction for transgenic mouse embryos

Transgenic mouse embryos at E11.5 were minced and enzymatically digested using Trypsin/EDTA at room temperature (RT) for 10 min. After the trypsinization, 2.0 × 10^6^ cells were collected and rinsed with 1% BSA/PBS. The cells were suspended in Cell Multiplexing Oligo (CMO) and incubated at RT for 5 min. Single-cell suspensions of embryos, which passed through a 40-μm cell strainer, were loaded onto FACSMelody Fusion (BD Biosciences) to remove debris. FACS-sorted cells were processed with Chromium Next GEM Single Cell 3′ Kit v3.1 (10× Genomics, Pleasanton, CA, USA) to construct a cDNA library, in accordance with the manufacturer’s instructions. The library, which was constructed using Dual Index TT Set A (PN-1000215; 10× Genomics), was sequenced on the NextSeq 2000 (Illumina, San Diego, CA, USA) sequencing platform using paired-end, dual-index sequencing.

To construct a transgene-specific cDNA library, the cDNA library obtained at step 2.3 in the manufacturer’s protocol was amplified to add adaptor sequences using PrimeSTAR MAX DNA Polymerase (Takara). The primer sequences for the transgene-specific scRNA-seq library are shown in Table S7. The library was again sequenced on NextSeq 2000 as described above.

#### Single-cell RNA-seq analysis for transgenic mouse embryos

The reads were mapped and aligned to the mouse reference genome (GRCm38) with EGFP sequences using the “cellranger multi” function of Cell Ranger Software v7.0.1 (10× Genomics). Downstream scRNA-seq data processing was performed using Seurat v5^65^. In brief, Soup X was performed to remove the effect of ambient RNA from the read count table.^66^ Because the clustering of cells and the dimensionality reduction, including uniform manifold approximation and projection (UMAP), should be affected by the transgene expression levels, we removed read count data of GFP before the normalization in the data processing step. scRNA-seq data with a high mitochondrial genome transcript ratio (≥5%), low ribosomal RNA transcript ratio (≤0.05%), and either low feature or UMI count (≤200) were excluded. Genes with UMI counts in fewer than three cells were also removed from this analysis. After this quality control, DoubletFinder was used to estimate and remove the doublets.^67^ To reduce the noise of scRNA-seq data, RECODE was adapted to our scRNA-seq dataset.^68^ For the pre-processed dataset, 2,000 highly variable genes were detected with the “FindVariableFeatures” function by the “vst” method using log-transformed normalized datasets (scale factor = 10,000). Principal component analysis (PCA) with variable genes was performed for dimensionality reduction. Cells were clustered and visualized by UMAP. The GFP expression values were added to the processed dataset without transgenes after UMAP visualization. The raw data is available at the GEO database (GSE306203).

For transgene analysis at the single-cell level, we analyzed the reads from a transgene-specific library. Each transgene-specific DNA BC sequence was extracted from the bam file, which was generated using the “cellranger multi” function. To remove error-derived barcodes, all DNA barcode pairs were compared. For each pair, we calculated Levenshtein distance (LD, equivalent to Hamming distance because lengths are equal) and read-count ratio: ratio = nlarge/nsmall where nlarge and nsmall are the read counts of the higher and lower abundance barcodes in the pair, respectively. If ratio >10LD (e.g., ≥10-fold for LD = 1, ≥100-fold for LD = 2), the lower count barcode was merged into the higher count barcode. This abundance-weighted clustering, iterated from the most frequent DNA barcode downward until convergence. After clustering, DNA barcodes supported by fewer than 100 reads were discarded. To mitigate PCR-recombination artifacts linking 10× barcode as cell ID to incorrect DNA barcode, read counts were tallied for every DNA barcode–10× barcode–UMI triplet. Counts were ranked, and an elbow point was identified with a custom script that selects the data point with the greatest perpendicular distance from the line connecting the first and last ranked points. To detect bona fide BC sequences, the fold change of the reads was calculated between each BC sequence using a read depth matrix ordered from top to bottom rank (related to Figure S6B). The cell types that expressed GFP at a low level were identified using the fold change of the normalized read number of the transgene (related to Figure S6E). Cell types below the abundance threshold were removed to evaluate the mosaicism quantitatively. Logarithmic read count data for each transgene based on DNA BC sequence were visualized as a heatmap using ComplexHeatmap (v2.20.0).^74^ The raw data is available at the GEO database (GSE309826).

#### BAC DNA recombination

A BAC DNA clone (RP24-125B24) containing the mouse Flk1 locus was obtained from Invitrogen (Carlsbad, CA, USA). Modification of the BAC DNA to insert the GFP sequence into the first exon of the Flk1 gene was performed in a previous study.^33^^,^^82^ To insert inverted terminal repeat (ITR) sequences and a Kusabira–Orange expression cassette into the backbone vector region, the BAC DNA was further modified by the same method as described in that previous study^33^ using the RED/ET recombination technique (Gene Bridges, Heidelberg, Germany). Collected recombinants were identified by screening for kanamycin resistance, followed by PCR analysis. The PGK-gb2-neo expression cassette was not excised.

#### Establishment of CdEC flox mouse line

We selected a sequence (5′-CGC CCA TCT TCT AGA AAG AC-3′) located in the first intron of the Gt(ROSA)26Sor as the CRISPR target. Briefly, an expression cassette consisting of CAG promoter, floxed EGFP, and Cas9NmC was placed between the 5′-homology arm and the 3′-homology arm. A rabbit β-globin polyadenylation signal sequence was placed downstream of each of floxed EGFP and Cas9NmC. Cas9NmC is a fusion protein of Cas9 and part of mouse Cdt1 that we previously reported.^83^ The CRISPR–Cas9 ribonucleoprotein complex and donor DNA were microinjected into zygotes of C57BL/6J mice, in accordance with our previous report.^84^ Subsequently, microinjected zygotes were transferred into the oviducts of a pseudopregnant ICR female and newborns were obtained. Genotyping for confirmation of the KI allele and random integration allele was performed using the same methods as in our previous report.^83^ The CdEC flox mouse line was deposited at RIKEN BioResource Research Center (RBRC12114).

#### Echocardiography

A Vevo 2100 High-Resolution Imaging System (Visual Sonics Inc., Toronto, Canada) was used to evaluate cardiac contractility. Mice were anesthetized with 2.5% isoflurane in oxygen and placed on a warmed platform. The isoflurane concentration was maintained at 1.5% during imaging. An ultrasound gel was applied to the left anterior thorax after the removal of fur from the body. Short-axis M-mode images were obtained at the level of the papillary muscle using a 40 MHz transducer. Left ventricular end-diastolic diameter (LVEDD) and left ventricular end-systolic diameter (LVESD) were measured using Vevo LAB software (Visual Sonics Inc.). Fractional shortening (FS) was calculated at three different time points for each mouse as follows: FS (%) = (LVEDD – LVESD)/LVEDD × 100.

### Quantification and statistical analysis

The data are presented as the mean ± values SD (Figures 1F, 2C, 3L, 4C, and 4J), mean ± SEM (Figure 5F) and as boxplots showing the median and interquartile range (Figure S3F). Statistical analyses were performed using ordinary one-way ANOVA (Figure 1F) or Mann-Whitney test (Figure 4J) with GraphPad Prism 10.4.2 (GraphPad software). *p* < 0.05 was considered statically significant.
