# Supplementary material for: Highly efficient and low-mosaicism piggyBac transgenesis platform for rapid founder phenotyping
Source: iScience. 2026 Jul 6;29(7):116648. doi: 10.1016/j.isci.2026.116648 (PMC13351448; doi:10.1016/j.isci.2026.116648)
Supplement: Document S1. Figures S1–S8 and Tables S1–S7 [file mmc1.pdf]

iScience, Volume *29*

Supplemental information

Highly efficient and low-mosaicism *piggyBac* transgenesis platform for rapid founder phenotyping

Eiichi Okamura, Shoma Matsumoto, Eiji Mizutani, Kazuya Murata, Yoko Tanimoto, Tra Thi Huong Dinh, Hayate Suzuki, Akihiro Kuno, Woojin Kang, Natsuki Mikami, Tomoka Ema, Kento Morimoto, Kanako Kato, Tomoko Matsumoto, Nanami Masuyama, Yusuke Kijima, Genta Nagae, Masanaga Muto, Toshifumi Morimura, Hideto Mori, Fumihiro Sugiyama, Satoru Takahashi, Hiroyuki Aburatani, Knut Woltjen, Nozomu Yachie, Seiya Mizuno, and Masatsugu Ema

Figure S1: Comparison of transgenic efficiency between hyPBase and mPBase

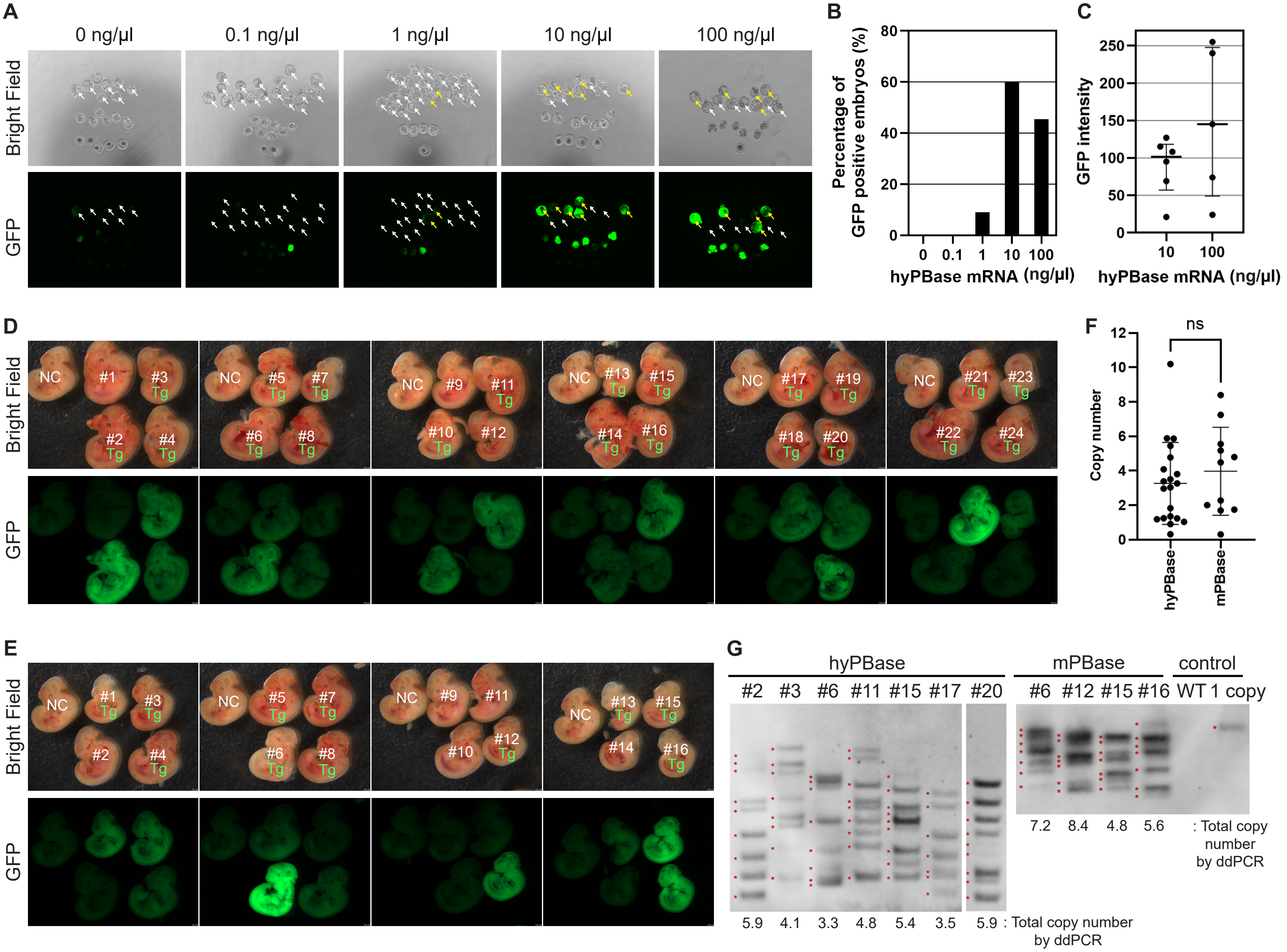

(A) Determination of the optimal hyPBase mRNA concentration. Fertilized eggs were electroporated with hyPBase mRNA (0, 0.1, 1, 10, or 100 ng/µl), followed by pronuclear microinjection of a donor vector encoding GFP under the CAG promoter. Embryos were cultured in vitro, and bright-field and GFP fluorescence images were acquired at E4.0. Scoring criteria are described in (B).

(B) Bar graph showing the percentage of Tg blastocysts at each hyPBase mRNA concentration. Weak GFP signals observed in the absence of hyPBase (0 ng/µl) represent background signals arising from transposase-independent random integration and/or transient expression of the donor DNA. Tg blastocysts were scored based on GFP intensity above background (yellow arrows, GFP-positive blastocysts; white arrows, background-level/GFP-negative blastocysts). Embryos without arrows were developmentally arrested or dead and were excluded from quantitative evaluation. Higher Tg rates were observed at 10 and 100 ng/µl hyPBase mRNA.

(C) Comparison of GFP fluorescence intensity in Tg embryos obtained with 10 vs 100 ng/µl hyPBase mRNA. GFP intensity was quantified by image analysis and plotted as the median with interquartile range (IQR). The highest GFP intensity was observed under the 100 ng/µl hyPBase mRNA condition.

(D, E) Fluorescence imaging of E11.5 embryos generated using 100 ng/µl hyPBase mRNA (D) or 500 ng/µl mPBase mRNA (E). Embryos positive for the GFP sequence by genotyping PCR are labeled “Tg”; negative controls are labeled “NC”.

(F) Transgene copy number per Tg embryo estimated by ddPCR.

(G) Southern blot analysis of Tg embryos. Transgene-specific bands are indicated by asterisks, and ddPCR-estimated copy numbers are shown below each lane. Note that hyPBase- and mPBase-derived samples were run on separate gels; therefore, band migration positions are not directly comparable between the two blots.

Figure S2: Generation of Tg embryos from PWK and B6 strains

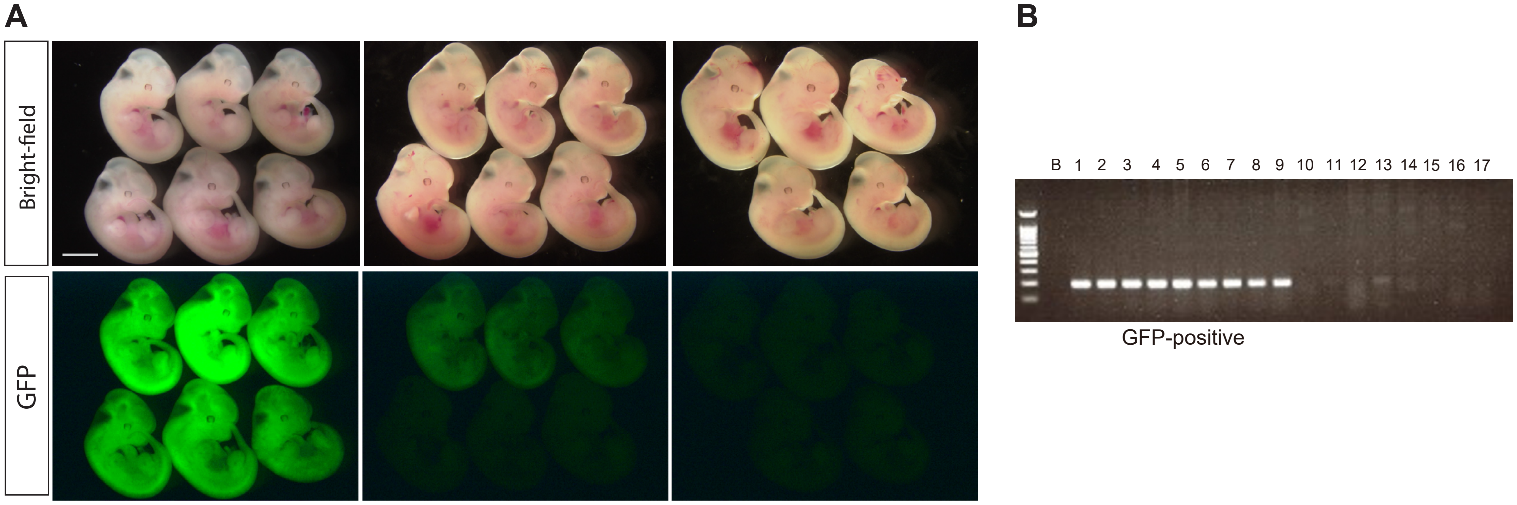

(A) E11.5 embryos generated using the optimized piggyBac system. CAG-GFP donor DNA with a barcode was introduced into fertilized eggs in the presence of mPBase mRNA, and the embryos were examined at E11.5. Scale bar = 2 mm.
(B) PCR genotyping of the embryos.

Figure S3: Generation of Tg embryos using the modified ICSI-Tr method

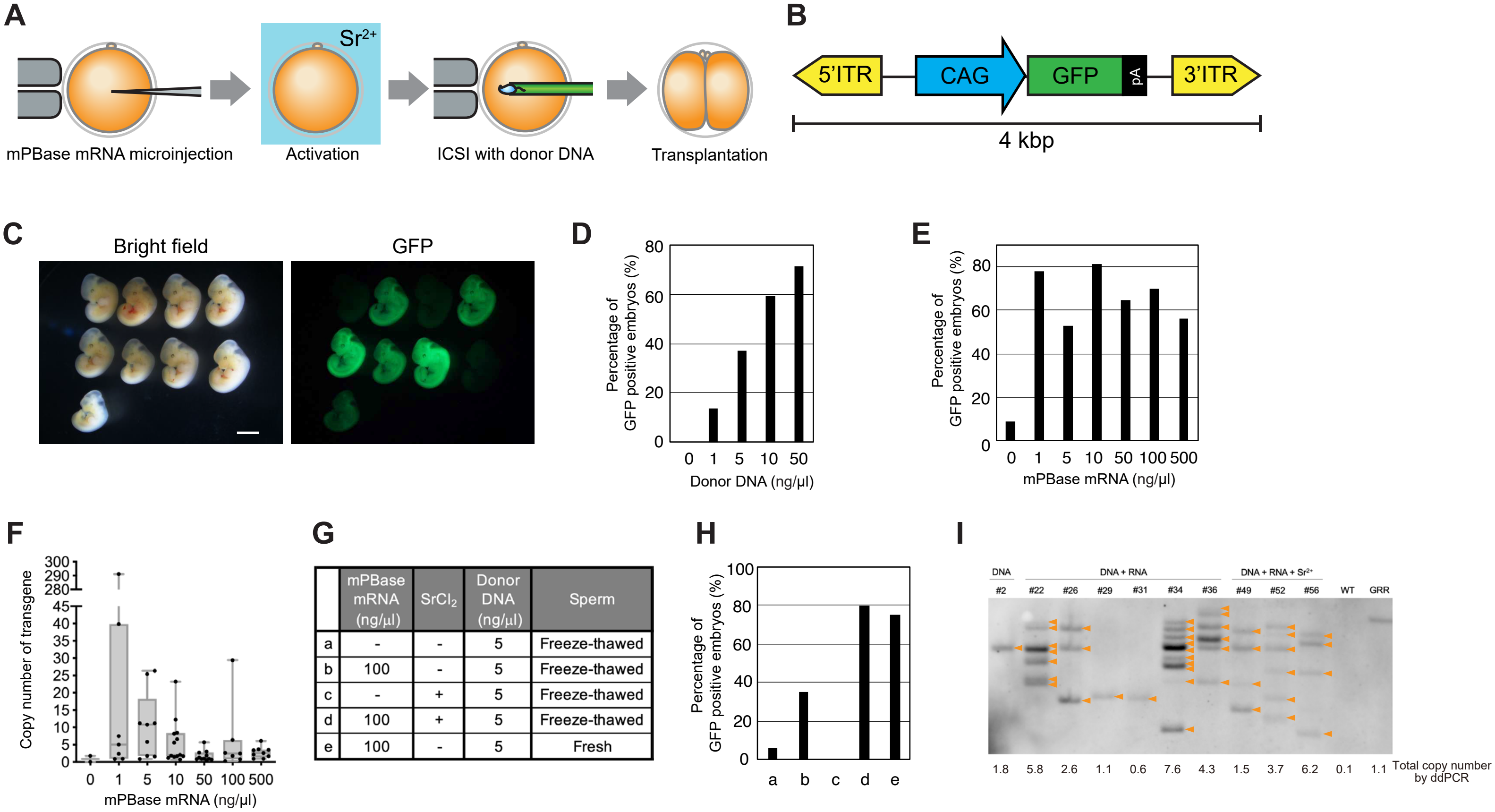

	mPBase mRNA (ng/µl)	$SrCl_2$	Donor DNA (ng/µl)	Sperm
a	-	-	5	Freeze-thawed
b	100	-	5	Freeze-thawed
c	-	+	5	Freeze-thawed
d	100	+	5	Freeze-thawed
e	100	-	5	Fresh

(A) Schematic representation of the method used to introduce mPBase mRNA and donor plasmid DNA by the modified ICSI-Tr method.

(B) Schematic representation of CAG-EGFP-BC donor plasmid DNA. Only the region between two ITRs is described.

(C, D) Investigation of the optimal concentration of donor DNA using DNA from E11.5 embryos. Images of Tg embryos obtained using 10 ng/µl donor DNA are shown in (C). Bright field (left) and GFP fluorescence (right). Scale bar: 5 mm. GFP-positive rates are displayed as a bar graph in (D). Blue dots indicate E11.5 development rate for the number of transplanted two-cell-stage embryos.

(E) Investigation of the optimal concentration of mPBase mRNA using DNA from E11.5 embryos. Transgenes were detected by conventional PCR and GFP-positive rates are displayed as a bar graph. Blue dots indicate E11.5 development rate for the number of transplanted two-cell-stage embryos.

(F) Investigation of the optimal concentration of mPBase mRNA using DNA from E11.5 embryos. Transgene copy numbers of Tg embryos were determined by ddPCR for the GFP sequence and are shown as a box plot.

(G) Experimental groups were compared to determine the optimal conditions of modified ICSI-Tr. A donor plasmid harboring 5.1 kbp of CAG-GFP was used for the modified ICSI-Tr method.

(H) Tg embryo ratio under each condition shown in (G). Transgenes were detected by conventional PCR.

(I) Southern blotting for GFP in E11.5 Tg embryos generated by ICSI-Tr. WT mouse and GRR KI mouse were used as negative and single copy controls, respectively.

Figure S4: Establishment of a quantitative method to identify the number of genome-integrated transgenes

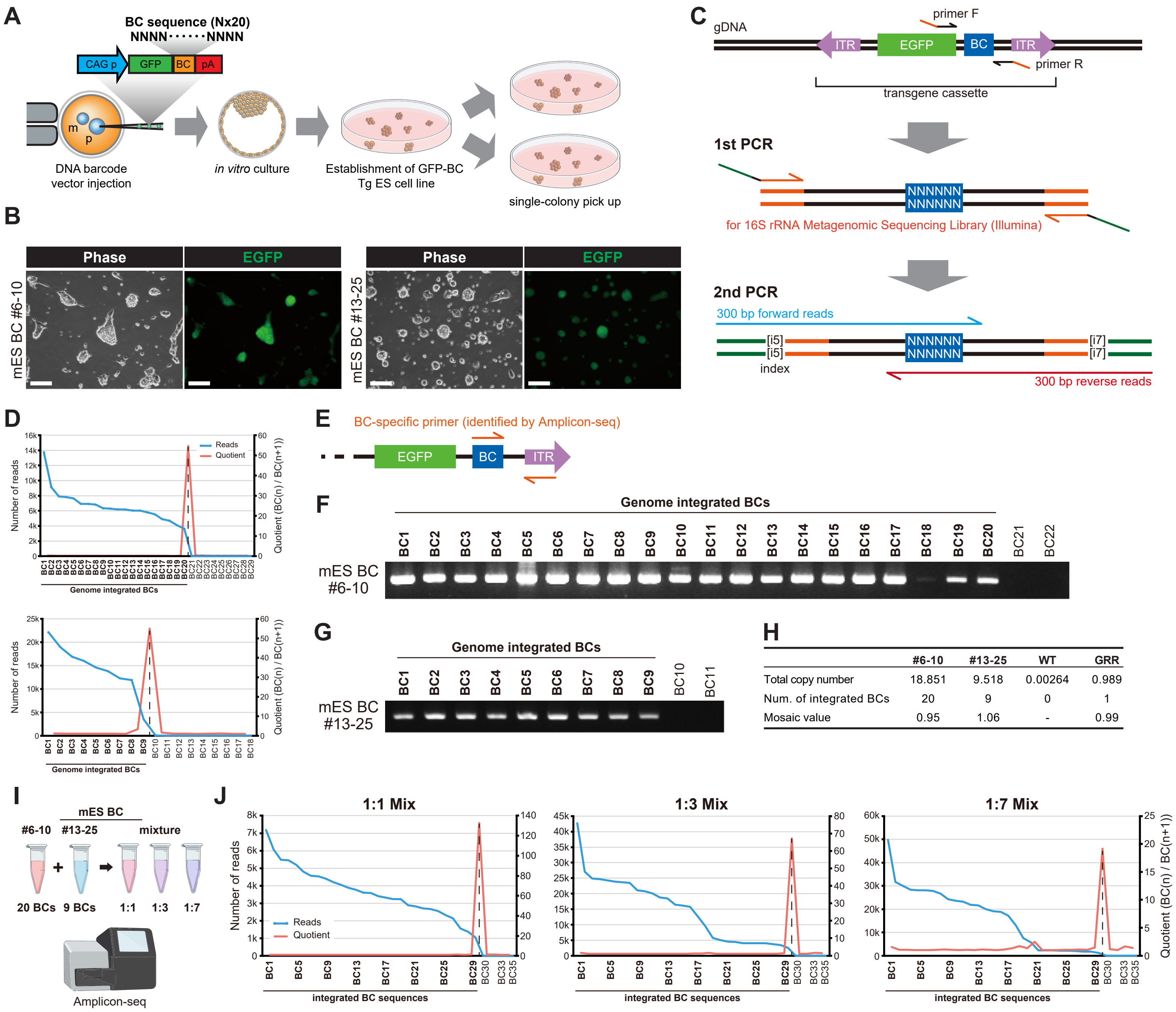

(A) Establishment of mESC lines possessing multiple BC sequences (mES BC) in their genome.

(B) GFP expression in cloned mESC lines possessing several copies of the EGFP-BC construct. Scale bar = 100 µm.

(C) A scheme showing construction of the Amplicon-seq library. BC region was amplified with index sequences for multiplexing.

(D, J) Detection of integrated BCs by Amplicon-seq. Blue line shows the read number for each BC sequence. Red line represents the fold change of the reads compared with that of the next rank of BC reads. The threshold of integrated BC detection is shown by the dashed line.

(E) BC-specific primers were used to validate integrated BCs identified by Amplicon-seq.

(F, G) BC-specific PCR for mES BC#6–10 and #13–25.

(H) The mosaic value was calculated from the total copy number by ddPCR and integrated transgene number by Amplicon-seq.

(I) Estimation of the mosaicism detection power by Amplicon-seq. Two cloned mES BC lines were mixed at ratios of 1:1, 1:3, and 1:7.

(J) Detection of integrated BCs for cloned mESC mixture samples by Amplicon-seq.

Parts of this figure were created in BioRender. Okamura, E. (2026) https://BioRender.com/6lepdkn.

Figure S5: Cell-type annotation of scRNA-seq

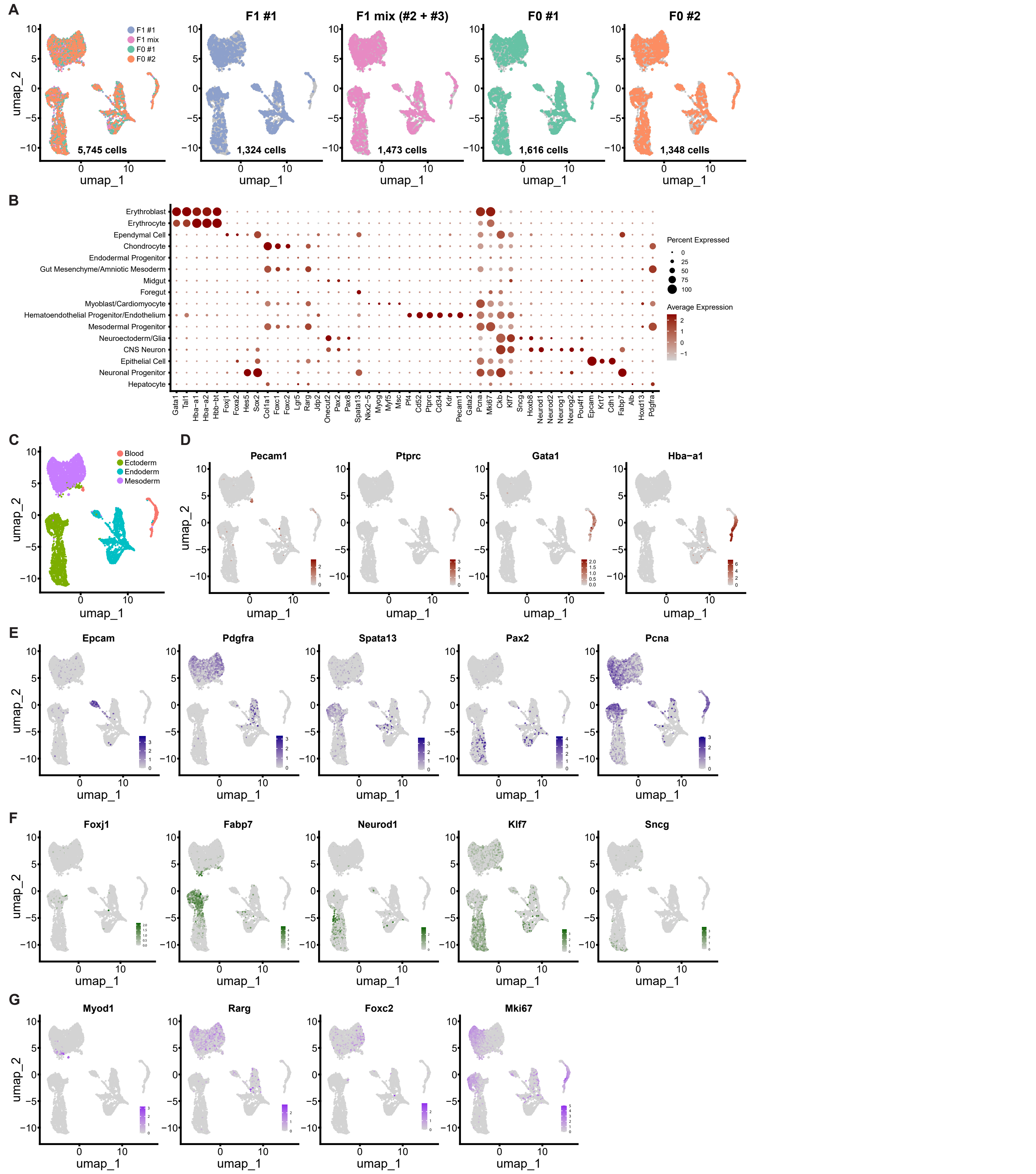

(A) UMAP of E11.5 Tg embryos. Each color shows a demultiplexed CellPlex sample.

(B) Bubble plot with each cell lineage marker. Color scale shows the average gene expression in each cell type and the size of the dot represents the proportion of the population with marker genes in the cell cluster.

(C) UMAP annotated with major cell lineage.

(D–G) Representative expression of the marker genes in each cell type derived from (D) blood, (E) endoderm, (F) ectoderm, and (G) mesoderm.

Figure S6: Quantitative transgene analyses for in vivo samples

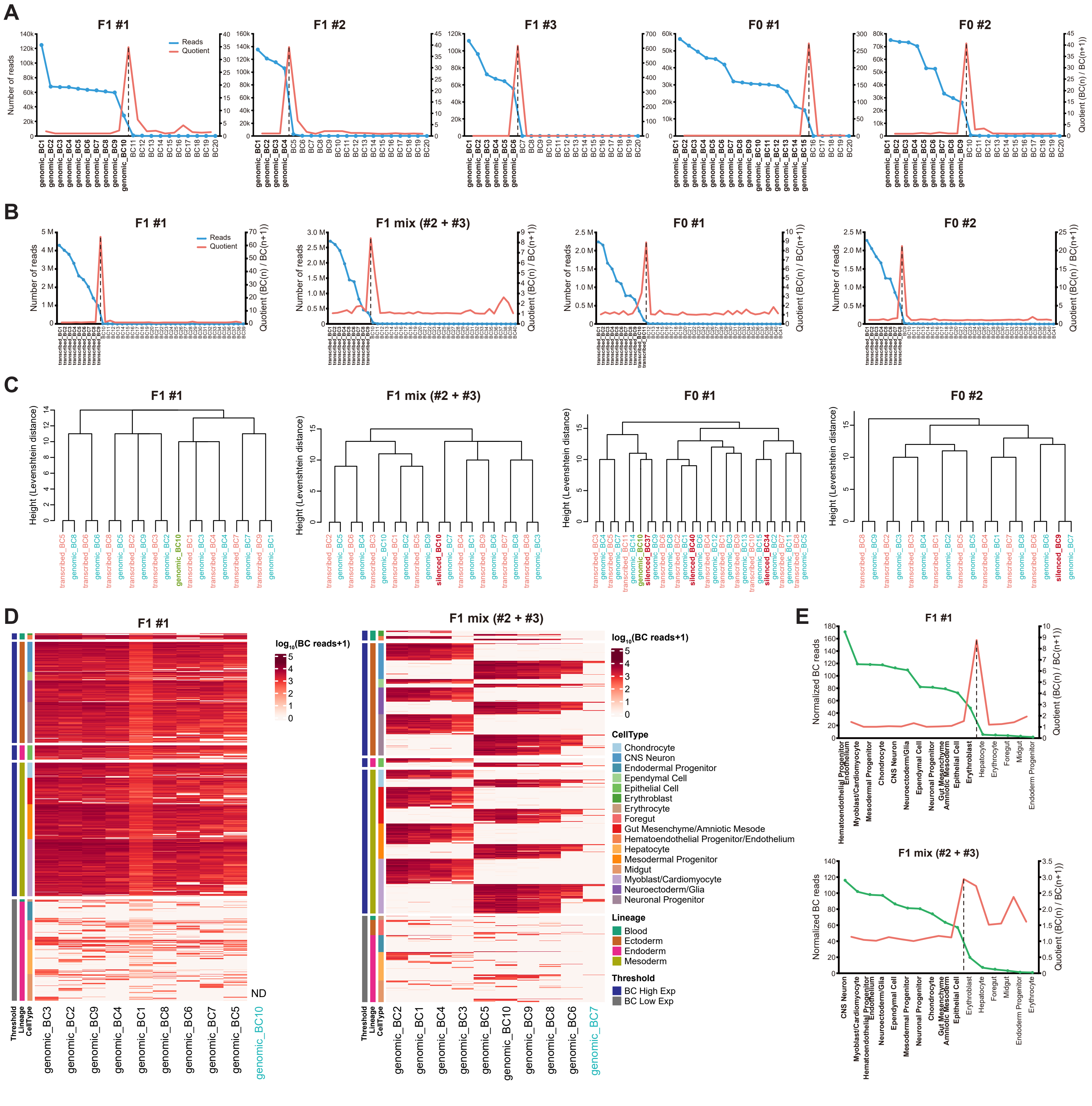

(A, B) Detection of (A) genomic and (B) transcribed transgenes identified by Amplicon-seq and transgene-specific scRNA-seq, respectively. Blue line shows the read number for each BC sequence. Red line represents the fold change of the reads compared with that of the next rank of BC reads. The threshold is shown by a dashed line.

(C) The cluster dendrograms of BC sequences for each transgene. The difference of BC sequences is shown using Levenshtein distance. Genomic and transcribed BCs are highlighted in blue and pink, respectively. The BCs identified only in genomic analysis are highlighted in green, and silenced transcribed BCs are shown in red.

(D) The heatmap shows the expression levels of individual genomic transgenes for all cell types in F1 #1 and F1 mix samples. Expression of each transgene was distinguished by the BC sequence. Cell type and cell lineage are shown by the color bars on the left of the heatmap. The cell types with low Tg expression are also shown by the color bar.

(E) Determination of the threshold for low-transgene-expression cell types caused by promoter activity. Green line shows the read number for each BC sequence. Red line represents the fold change of the reads compared with that of the next rank of BC reads. The threshold is shown by a dashed line.

Figure S7: CRISPResso2 analysis showing base-by-base agreement with the reference sequence

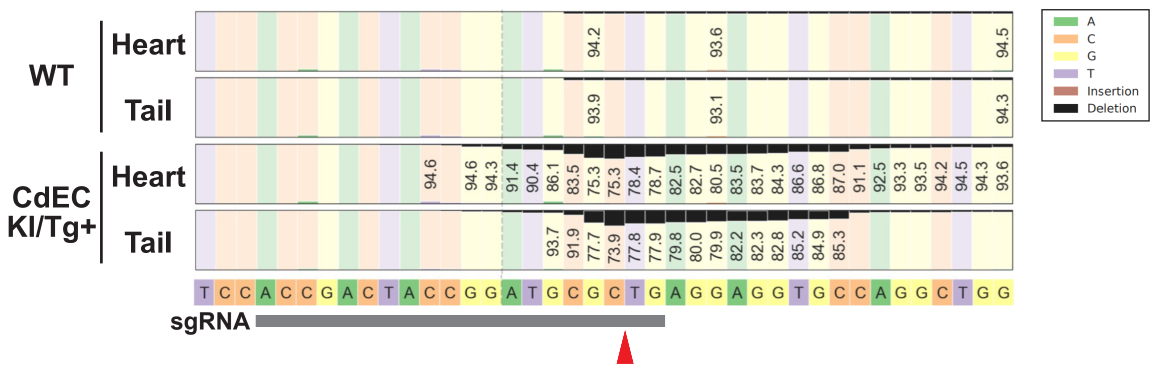

Amplicon sequencing reads were analyzed using CRISPResso2. Results are shown for WT and CdEC KI/Tg+ animals from heart and tail tissues. Colored boxes indicate the reference amplicon sequence, and the sgRNA target region is shown below. Values on the plot represent the percentage of reads matching the reference base at each nucleotide position. The red arrowhead indicates the predicted nucleotide cleavage site.

Figure S8: Assessment of genome editing at predicted off-target sites

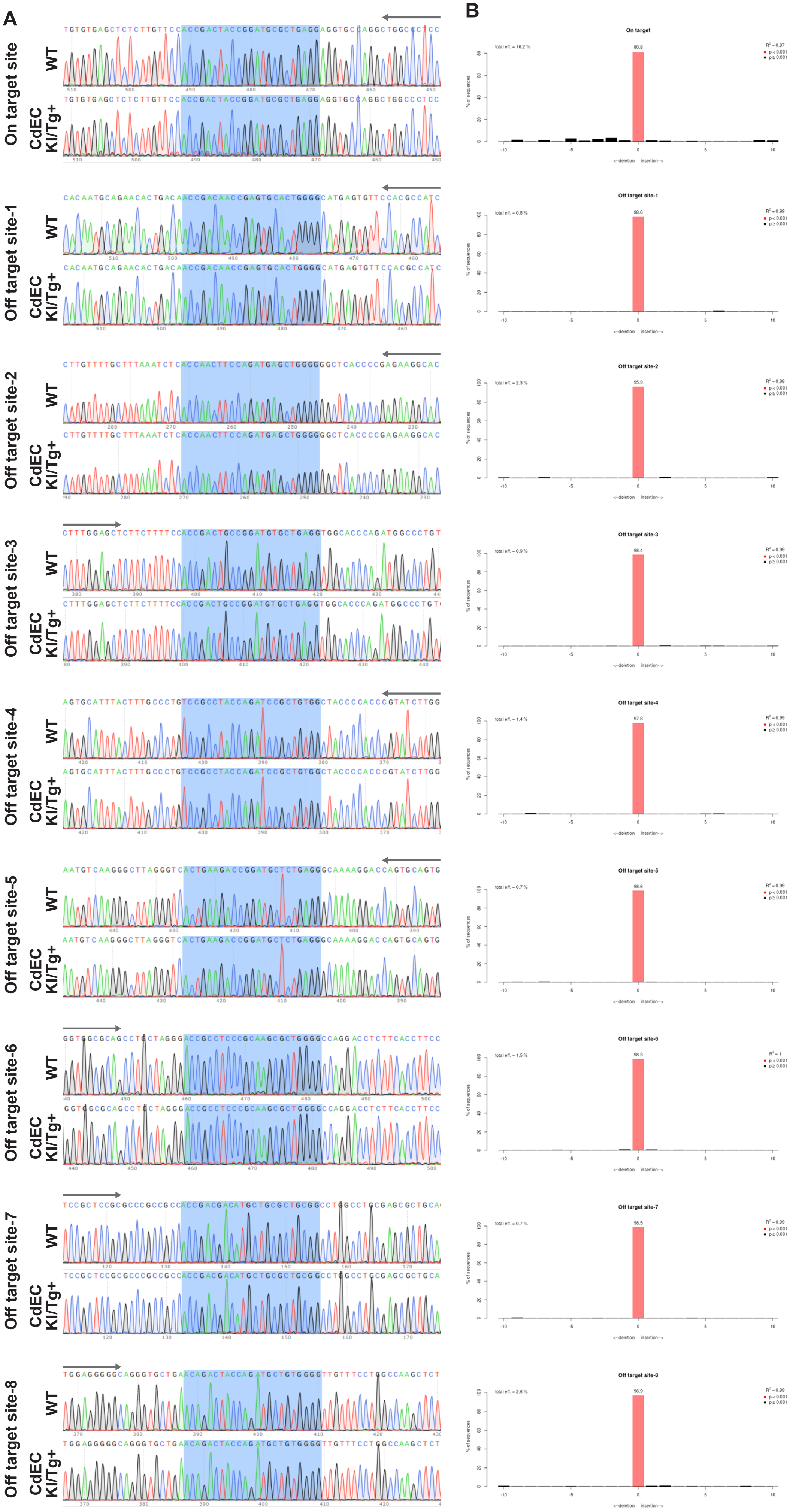

(A) Sanger sequencing chromatograms of the on-target locus and eight predicted off-target sites. Genomic DNA was isolated from heart tissue of WT and CdEC KI/Tg+ mice and PCR-amplified across the on-target site and each off-target candidate site. The sgRNA-binding region is highlighted in blue. Arrows indicate the sequencing direction.

(B) TIDE analysis of the Sanger traces shown in (A). Chromatograms from a WT mouse were used as the control sample, and chromatograms from a CdEC KI/Tg+ mouse were used as the test sample. Indel spectra and estimated editing frequencies are shown.

Table S1: Previous reports of transgenesis by piggyBac transposase in mice

Year	Mouse strain	Transposase			Donor DNA		Tg ratio (%, /animals)	Journal	PMID
		Type		Delivery method	Type	Delivery method			
2025	B6D2F1	hyPBase	mRNA	ICSI coinjection	Plasmid	ICSI coinjection	62	Nat Commun.	40128182
2023	B6D2F1	hyPBase	mRNA	Cytoplasmic injection	Plasmid	Cytoplasmic injection	37 - 56	Epigenetics & Chromatin	36522780
2021	B6D2F1	hyPBase	mRNA	Pronuclear injection	Plasmid	Pronuclear injection	3.5 - 8.3	Sci Rep.	34083640
2014	B6CBAF1/J	hyPBase	mRNA	Pronuclear injection	BAC	Pronuclear injection	25 - 50	Dev Dyn.	24924516
2014	Kunming white mouse	PBase*	Plasmid	Pronuclear injection	Plasmid	Pronuclear injection	4.2	Amino Acids	24893662
2013	C57BL/6J	hyPBase	mRNA	Pronuclear injection	BAC	Pronuclear injection	22 - 45	Genesis	23225373
2013	B6D2F1	mPBase	mRNA	Pronuclear injection	Plasmid	Pronuclear injection	18 - 21	FASEB J.	23195032
2012	B6D2F1	hyPBase	Plasmid	ICSI coinjection	Plasmid	ICSI coinjection	0 - 100	Proc Natl Acad Sci U S A.	23093669
2010	FVB/NJ	PBase	mRNA	Pronuclear injection	Plasmid	Pronuclear injection	10 - 46	PLoS One	21179568
2010	B6D2F1	PBase, mPBase	Plasmid	ICSI coinjection	Plasmid	ICSI coinjection	11.5 - 69.2	Proc Natl Acad Sci U S A.	20404201
2005	FVB/NJ	PBase	Plasmid	Pronuclear injection	Plasmid	Pronuclear injection	34.8 - 65	Cell	16096065

*not explicity specified

Table S2: Experimental conditions and outcomes for transgenic mouse production by hyPBase and mPBase

Figure	Materials									Results								
	Mouse strain		Transposase	mRNA Conc. (ng/ul)	Donor DNA					Electro-porated	Survived (% of electro-porated)	Micro-injected	Survived (% of micro injected)	Developed to 2 cell stage	Blastocysts	Trans-planted	E11.5 embryo	Transgenic (Ratio)
	Male	Female			Construct	Vector type	Total length (kbp)	Insert length (5'ITR to 3'ITR) (kbp)	Conc. (ng/ul)									
Supplemental figure S1A	B6N	B6D2F1	hyPBase	0	CAG-GFP	Plasmid	8.1	5.1	5	20	19 (95%)	19	14 (74%)	-	8	-	-	0 (0%)
				0.1						30	27 (90%)	27	25 (93%)	-	17	-	-	0 (0%)
				1						30	27 (90%)	27	26 (96%)	-	22	-	-	2 (9%)
				10						30	21 (70%)	21	17 (81%)	-	10	-	-	6 (60%)
				100						30	18 (60%)	18	17 (94%)	-	11	-	-	5 (46%)
Supplemental figure S1D	B6J	B6J	hyPBase	100	CAG-GFP	Plasmid	7.0	4.0	4.3	250	219 (88%)	174	154 (89%)	142	-	133	24	20 (83%)
Supplemental figure S1E	B6J	B6J	mPBase	500	CAG-GFP	Plasmid	7.0	4.0	4.3	120	117 (98%)	94	89 (95%)	84	-	48	16	11 (69%)

Table S3: Summary of transgenic mice production experiments by ICSI-Tr method using mPBase

Figure	Materials										Results							
	Mouse strain		Sperm	PBase mRNA Conc. (ng/ul)	Act (St+)	Donor DNA					mRNA injection	Act (St+)	ICSI	PN	2-cell	Trans-planted	E11.5 embryo (%)	Transgenic (%)
	Male	Female				Construct	Vector type	Total length (kbp)	Insert length (5'ITR to 3'ITR) (kbp)	Conc. (ng/ul)								
Supplemental Fig. S3D	B6N	BDF1	Fresh	100	+	pNM1419	Plasmid	7.0	4.0	0	84	83	80	80	78	67	36 (53.7%)	0 (0%)
					+					1	78	74	73	73	73	48	22 (45.8%)	3 (13.6%)
					+					5	111	110	109	109	97	67	27 (40.2%)	10 (37.0%)
					+					10	79	76	76	76	58	51	22 (43.1%)	13 (59.1%)
					+					50	110	101	98	97	47	41	14 (34.1%)	10 (71.4%)
Supplemental Fig. S3E	B6N	BDF1	Fresh	0	+	pNM1419	Plasmid	7.0	4.0	10	70	60	55	55	49	49	22 (44.9%)	2 (9.1%)
				1	+						72	57	52	51	46	27	9 (33.3%)	7 (77.8%)
				5	+						72	62	61	61	58	58	17 (29.3%)	9 (52.9%)
				10	+						72	63	63	61	55	55	16 (29.1%)	13 (81.3%)
				50	+						72	68	67	65	60	60	17 (28.3%)	11 (64.7%)
				100	+						73	66	64	64	54	48	10 (20.8%)	7 (70.0%)
				500	+						71	68	67	67	62	44	16 (36.3%)	9 (56.3%)
Supplemental Fig. S3H	B6N	BDF1	Freeze-thawed	-	+	CAG-GFP	Plasmid	8.1	5.1	5	N.A.	N.A.	29	N.A.	28	28	18	1 (5.5%)
				100	+								31		29	29	23	8 (34.8%)
				-	+								26		25	25	7	0 (0%)
				100	+								37		37	37	10	8 (80%)
			Fresh	100	+								24		21	21	4	3 (75%)

Table S4: Summary of transgenic mice production experiments using mPBase

Figure	Materials								Results											
	Mouse strain		PBase mRNA Conc. (ng/ul)	Donor DNA					Electro-porated	Survived (% of electro-porated)	Micro-injected	Survived (% of micro injected)	Developed to 2 cell stage	Trans-planted	Sampling					Transgenic (Ratio)
	Male	Female		Construct	Vector type	Total length (kbp)	Insert length (5'ITR to 3'ITR) (kbp)	Conc. (ng/ul)							Blastocyst	E9.5 embryo	E11.5 embryo	E14.5 embryo	After birth	
Fig.1D	B6J	B6J	0	CAG-GFP	Plasmid	8.1	5.1	5	88	87 (99%)	87	76 (87%)	76	-	13	-	-	-	-	0 (0%)**
			20						88	84 (96%)	84	72 (86%)	72	-	15	-	-	-	-	13 (87%)**
			100						88	84 (93%)	84	74 (88%)	74	-	16	-	-	-	-	8 (50%)**
			500						87	81 (92%)	81	77 (95%)	77	-	12	-	-	-	-	9 (50%)**
Fig.1E, F	B6J	B6J	0	CAG-GFP	Plasmid	8.1	5.1	5	65	60 (92%)	60	55 (92%)	53	44	-	-	10	-	-	0
			100						66	60 (92%)	60	60 (100%)	58	48	-	-	10	-	-	9 (90%)
			500						66	61 (92%)	61	58 (95%)	57	46	-	-	13	-	-	13 (100%)
			1000						65	60 (92%)	60	59 (98%)	53	44	-	-	11	-	-	11 (100%)
Fig.2	PWK	B6J	500	pNM1419	Plasmid	7.0	4.0	5	235	230 (98%)	186	168 (90%)	160	48*	-	-	17	-	-	9 (53%)
Fig.3	B6J	B6J	500	pNM1419	Plasmid	7.0	4.0	5	193	192 (100%)	122	116 (95%)	114	24	-	-	16	-	-	16 (100%)
Fig.4A	B6J	B6J	500	Ubc-GFP-mCherry	Plasmid	15.2	11.8	12	170	169 (100%)	141	130 (92%)	118	72	-	-	8	-	-	8 (100%)
Fig.4B	B6J	B6J	200	Myh6 pro.-GFP-CAG-tdTomato	Plasmid	14.7	12.4	9.1	240	240 (96%)	225	199 (88%)	144	30	-	2	-	-	-	2 (100%)
								5	477	449	126	121	90	22	-	-	7	-	-	7 (100%)
Fig.4J	B6J	B6J	500	*Flk1*-GFP	BAC	183	181	1	387	373 (96%)	161	150 (93%)	130	120	-	-	19	-	-	14 (74 %)
						183	181	10			161	149 (93%)	84	96*	-	-	10	-	-	7 (70 %)
Fig. 4F	B6J	B6J	500	pmPB-ITR tagBFP<br>pmPB-ITR EGFP<br>pmPB-ITR tdTomato	Plasmid	7.2<br>7.2<br>8.0	4.2<br>4.2<br>4.9	5, each	N.A.	N.A.	117	N.A.	-	103	-	-	-	12	-	11 (92%)**
	B6J	B6J	500	pmPB-ITR tagBFP<br>pmPB-ITR EGFP<br>pmPB-ITR tdTomato<br>pmPB-ITR E2Crimson	Plasmid	7.2<br>7.2<br>8.0<br>7.2	4.2<br>4.2<br>4.9<br>4.2	5, each	N.A.	N.A.	194	N.A.	-	169	-	-	-	4	-	3 (75%)**
Fig.5A−G	CdECflox (Homo)	B6J	500	Myh6-Cre-Prmt1	Plasmid	12.3	9.2	5	300	279 (93%)	263	249 (95%)	-	249***	-	-	-	-	10	1 (10%)
	GRR (Homo)								416	402 (97%)	402	377 (94%)	-	377***	-	-	-	-	14	10 (71%)
Fig.5H−J	CdECflox (Homo)	B6J	500	pBS-mPBITR-one gRNA-Ddx4p-CI-CreERT2	Plasmid	11.9	9.0	N.A.	N.A.	N.A.	72	N.A.	-	46	-	-	-	-	14	14 (100%)

* inclucing 1-cell stage and 2-cell stage embryos
** judged by fluorescence observation
***transplanted at 1-cell-stage
N.A.: Not available

Table S5: CRISPOR-predicted off-target sites for the Prmt1 gRNA and PCR primer sequences used for Sanger sequencing

No.	locus Description	Position	off-target Sequence	MIT Off-target Score	CFD Off-target Score	PCR primer (forward)	PCR primer (reverse)
1	intergenic:Slc30a10-Lyplal1	chr1:185619196-185619218:-	ACCGAC**A**ACCG**AG**TGC**A**CTG GGG	0.073	0.497	GACTACCCGGAGCACATCAG	CACATCCTGCCCACTCCTTT
2	intergenic:Rbm19-Gm26474	chr5:120210997-120211019:+	ACC**A**ACT**T**CC**A**GATG**A**GCTG GGG	0.143	0.386	ATGGCTGCGAGTGATTGACA	AGTGGTCACCTTGAGAAGCG
3	intergenic:Hmgb1-ps3-Hs3st3b1	chr11:63878879-63878901:+	ACCGACT**G**CCGGATG**T**GCTG AGG	1.297	0.226	TCGAGGACAAAAGTCACCGG	TCCCCAGTCCCTAAAACCCA
4	exon:Ctu2/Piezo1	chr8:122485034-122485056:-	**T**CCG**C**CTACC**A**GAT**C**CGCTG TGG	0.231	0.136	TCCGCTGTCCACAAGAGTTC	CACCCTGTCCTGTCCCATTC
5	intron:Gm872	chr10:92817067-92817089:+	AC**T**GA**AG**ACCGGATGC**T**CTG AGG	0.244	0.094	CTGGTAGGGGCAGAGAGAGT	ACGCAATACAGGATGCTTACTGA
6	intergenic:Eif1b-Entpd3	chr9:120539756-120539778:-	ACCG**C**CCT**C**CCG**C**A**A**GCGCTG GGG	0.105	0.070	GGCTGGGGTGAAGTGAAAGA	TATTCACAAGGCTCGGGCTG
7	exon:Perp	chr10:18845184-18845206:+	ACCGAC**G**AC**AT**G**C**TGCGCTG CGG	0.184	0.041	TCACCGGAATCAAGGTGTGG	TTGAGCCTGATTCGGGTCAC
8	intergenic:Nox3-Gm23186	chr17:4636784-4636806:-	AC**A**GACTACC**A**GATGC**TG**TG GGG	0.065	0.029	TTTCTAGGGCCCACAAGCAG	TGTCAATAGAGTTGGCCCCG

Table S6: DNA barcode sequence analysis by Sanger sequencing

No.	BC sequence	No.	BC sequence	No.	BC sequence	No.	BC sequence
1	AATGTGGGTATCTCGAGGCG	26	CTAGCGAGGCGTCCACAGAT	51	CACGACATGGAGACTATAGA	76	CACTATACGGCATACATCGT
2	CATGATGCCACCGGACGCCA	27	CACTATACGGCATACATCGT	52	CTTATTTTTCTATTAATGTT	77	CTCTAACACGATGCCCTGTC
3	CATATCCTCCTTACTATCCT	28	TCGCGTGCAAAATTGAGCAC	53	CTACCATTCCCGAGATCTGA	78	GCGTTCATATTGTGGGCACT
4	GCTATAGGGCTGCAGGATCT	29	TATTGATAAAAAGAAGGCGT	54	CAAATTAAGTTATCATCGTT	79	TTAGAAATCGGTGCTGAAAT
5	CGTTCAGTTGTGGTGTCGAT	30	AGAGTCAGCTTCGGCACTTG	55	TCGGGATCAAGGTTGTGATT	80	ATCAGTCCCTGATGGATTCT
6	CTCAGGAGTTATTGAGCAAG	31	CCTTTTGTATTGGGGGGATG	56	TATCAATCGCGCTTATATGG	81	TTCTAAAGGAGATGGTAGCG
7	TTGTACTTTTTTATAAATCT	32	CGGCTTTCTACCCATTTTTA	57	TTGTCACATAATCGTCGGCG	82	CCACAATTCAGATTCGCAGT
8	TCATTAACGTCGGTCTGTCG	33	AATTATCTGGGTGTTTGCGC	58	GTACGATATGTATAATGCCT	83	CTTCTCATAGCTCTTATTAG
9	TTAATGACCTTTTCTCTGCG	34	CGGTCCCGTTAATGGGGGGG	59	GCATTATTCAGCGAACAACT	84	TTCCATTGAATCCAACGACA
10	TTATCTCAAACGGACCTTCG	35	CACATGCCGAGGCTTGCTTC	60	GATTGTATCTAAAGTACACC	85	TATCCACACAAGGAAAGCCT
11	CCCCCCGAACCTTAGGTCGT	36	GGTCAGACATTTGCTGGTCC	61	TTAAGACCATCGGTAAGTAT	86	TTATGTAGCCGGCCAGTCGT
12	CAGGCACGGCTTTGGCGTTT	37	CAACGTACGTTTCCTGACTT	62	TGCATTGACCGAGCGTGGCG	87	AAATCTGGCGGGCGGGCACA
13	TCGACTCTCATATCCGAGTA	38	AAACACATTTGCACACACTT	63	AATCCAGTGTGAGGTTTCGA	88	CGGAAACCTGTATGAAGCTT
14	TATTAAGCTGTTCAAATTAT	39	CCATAATTAACGTCCCGGAC	64	TTAGAGAACCACTAATACTG	89	CGGTTCTCGCACTTACATGG
15	TAACTGAAGTGACAAGATCT	40	TACTTTTAACAGTCTCGTCG	65	CCGTCCAAAGCTCCCCAGAC	90	GCTATAGGACATATGTACTG
16	GTCACGTGTCATCGCTAATG	41	TGCTCCTGACCCGCTGTCC	66	GATTACGCCAACTTCTGTAT	91	CATGCGATAAAAAATTGAAT
17	TGGTTGTACTAGGCCGTCAA	42	AAGTCGTCCTTGTGATGTGT	67	TATAGCACAGCCAGCCTTGG	92	GTAGCGATTGAATATATATA
18	TATCTAATCTATATTACTTC	43	CTTTGACGGCACCGGGTATC	68	TGATCCCAGTTAATTGAGCG	93	ACATACAGTCAGTCTTTTGT
19	TTGCAGCATAAGTTTGTA	44	TGTGAGTAGAAAGATCATAC	69	GGGGACACAGTCGCCGTCCA	94	TCCGACCACCATTCCAGACT
20	TTCGTAAAAAGCGTTGATGG	45	CTAATCCTGCTGAACACTGG	70	CTCGCCCTACCTCAACGAGT	95	TCTCTAAATAGCTGGGCATG
21	CGGTCTAGGATGACGATTTG	46	TTTTATGTTTCTGTATCGCG	71	GCTTAGTTGGCCACGTATA	96	ACGCAAGGATAATTGTGTAT
22	ATTCATTGTTCGTCGGGCTC	47	ACGACTAGGACCTAGTCCTG	72	CGCTAGCATACCTACCCCACA	97	TCAGTTGAGTTTTAGTCCGA
23	GTTTAAGATTCATAGTGATC	48	TGGTATCGGACTCTTTATGG	73	ACGCCCTTATCTTCTGCACC	98	GTTCGAGTTCTACAAAGTCT
24	AATTGTCCTCGTATCTCGGG	49	ATCGCCCCCTTGACGTAAAG	74	TATAATTCAGGATGGTACGT		
25	CATTGAGTATGCCAGGCCTG	50	GAATTCTAGAGGTTACTTT	75	AACTTTCTACTGGGATTTAC		

Duplicated

Table S7: Primer sequences

Name	Seq	Use
BC-fragment-F	CTCGAGCCTCTAGATTCTGCAGCCCTATAGCACCGGTNNNNNNNNNNNNNNNNNNNNCAGGACCTAAAGAATCCCAT	DNA barcode fragment
BC-fragment-R	GGGTCGTGGGGCGGGCGACGCGTGGATGGGATTCTTTAGGTCCTG	DNA barcode fragment
GFP_F	AAGTCGTGCTGCTTCATGTG	Genotyping PCR
GFP_R	ACGTAAACGGCCACAAGTTC	Genotyping PCR
Rbm31_F	CACCTTAAGAACAAGCCAATACA	PCR_control
Rbm31_R	GGCTTGTCCTGAAAACATTTGG	PCR_control
GFP-probe_FAM_ddPCR	ACGACGGCAACTACA	ddPCR
GFP_primer_F_ddPCR	GAGCGCACCATCTTCTTCAAG	ddPCR
GFP_primer_R_ddPCR	TGTCGCCCTCGAACTTCAC	ddPCR
pNM1419_Inverse_F	GCTAGCCGAGAGCATAATATTGATATG	pNM1419 inverse-PCR 1st PCR
pNM1419_Inverse_R	CCCTGAACCTGAAACATAAAATGAAT	pNM1419 inverse-PCR 1st PCR
pNM1419_Nested_F	AAATTAATAAATAAACCTCGATATACAGA	pNM1419 inverse-PCR 2nd PCR
pNM1419_Nested_R	TCATGTCTGGATCTACTGGGGT	pNM1419 inverse-PCR 2nd PCR
pNM1419_integrated_F	CCGATAAAACACATGCGTCAAT	pNM1419 inverse-PCR 3rd PCR, Validation for pNM1419 inverse-PCR
PWK1_BC1_R	ATAAACACCCTAAACACCCA	pNM1419 inverse-PCR 3rd PCR
PWK1_BC2_R	ACCACAAACAAATCCCAAAA	pNM1419 inverse-PCR 3rd PCR
PWK1_BC3_R	ACAAAACCGACCACCTAAAA	pNM1419 inverse-PCR 3rd PCR
PWK2_BC1_R	GAAAAATACAATCAAACCAC	pNM1419 inverse-PCR 3rd PCR
PWK2_BC2_R	AAACAACAAAAGCCAAATAA	pNM1419 inverse-PCR 3rd PCR
PWK3_BC1_R	CAAAACACAAAAAAGGGCAC	pNM1419 inverse-PCR 3rd PCR
PWK4_BC1_R	ACCAGGAAATCCACCCAAAC	pNM1419 inverse-PCR 3rd PCR
PWK5_BC1_R	AACTAACCCGAGAAGAACAA	pNM1419 inverse-PCR 3rd PCR
PWK5_BC2_R	CAAAAGAAGAAAACCAGCAA	pNM1419 inverse-PCR 3rd PCR
PWK6_BC1_R	TCACAACACAGCAAGACACA	pNM1419 inverse-PCR 3rd PCR
PWK6_BC2_R	CACAAAATAAAATAAGCCAC	pNM1419 inverse-PCR 3rd PCR
PWK6_BC3_R	AACCCTAACAGGAGCAAAAA	pNM1419 inverse-PCR 3rd PCR
PWK7_BC1_R	AAGAGTAAACAACAGGCACA	pNM1419 inverse-PCR 3rd PCR
PWK7_BC2_R	AGACACGAATAACACGAAAC	pNM1419 inverse-PCR 3rd PCR
PWK8_BC1_R	CACAGCAACAGACACCAAAAAAAAA	pNM1419 inverse-PCR 3rd PCR
PWK9_BC1_R	CCCCAAACACTCAACTAACC	pNM1419 inverse-PCR 3rd PCR
pNM1419_Inverse_sequencing	AACGTACGTCACAATATGATTATCT	Sequencing primer for inverse-PCR
pNM1419_5'ITR_R	GAGAGCAATATTTCAAGAATGC	Validation for pNM1419 inverse-PCR
PWK1_BC1_gt_F	CAGCAGAGCATGGCAACACA	Validation for pNM1419 inverse-PCR
PWK1_BC1_gt_R	CCCGGCGTTTCAACAAGAAC	Validation for pNM1419 inverse-PCR
PWK1_BC2_gt_F	TCAGTGTGGCAGGGCCAATA	Validation for pNM1419 inverse-PCR
PWK1_BC2_gt_R	GCAGTGCAGGAAAAGGGTCA	Validation for pNM1419 inverse-PCR
PWK1_BC3_gt_F	AGCAAGGCTGCTGGTGGAAG	Validation for pNM1419 inverse-PCR
PWK1_BC3_gt_R	ACCAGGGCAAGCAAATGGAA	Validation for pNM1419 inverse-PCR
PWK2_BC1_gt_F	AAGCACCCATGTTTGCAAGTGA	Validation for pNM1419 inverse-PCR
PWK2_BC1_gt_R	CGGCTGTCCTGGCTCAAACT	Validation for pNM1419 inverse-PCR
PWK3_BC1_gt_F	ATGCTTTGGCCCTGCTTGAC	Validation for pNM1419 inverse-PCR
PWK3_BC1_gt_R	AGCCACACAGCGAAGCACAC	Validation for pNM1419 inverse-PCR
PWK4_BC1_gt_F	TGAAAGGCGTGCACCACACT	Validation for pNM1419 inverse-PCR
PWK4_BC1_gt_R	AGACACCCCCTGGCTGCAT	Validation for pNM1419 inverse-PCR
PWK5_BC1_gt_F	CCCTGTGTTCTATGCTTCGCACT	Validation for pNM1419 inverse-PCR
PWK5_BC1_gt_R	CGATGGTGTTTTGCCTGCAT	Validation for pNM1419 inverse-PCR
PWK5_BC2_gt_F	TTCCACGCGAAAACCTGGAT	Validation for pNM1419 inverse-PCR
PWK5_BC2_gt_R	TTCCTGCTCCTCCCAGCATC	Validation for pNM1419 inverse-PCR
PWK6_BC1_gt_F	TGGCACAGGTGCAGCATTTT	Validation for pNM1419 inverse-PCR
PWK6_BC1_gt_R	GATGCCCAGGCTCATTCCTG	Validation for pNM1419 inverse-PCR
PWK6_BC3_gt_F	CAGGGAAGGGGAAAGGGCTA	Validation for pNM1419 inverse-PCR
PWK6_BC3_gt_R	TTCCTGCCCTGCTGGAGTTC	Validation for pNM1419 inverse-PCR
PWK7_BC1_gt_F	TCCCACAGTTTGGTCTCTCACAA	Validation for pNM1419 inverse-PCR
PWK7_BC1_gt_R	CAGTGTGCAGAGGTAGCAAAGGAA	Validation for pNM1419 inverse-PCR
PWK8_BC1_gt_F	GGCTGGCCTCGAACTCAGAA	Validation for pNM1419 inverse-PCR
PWK8_BC1_gt_R	AGCCGGAAGAGCCGTCTACA	Validation for pNM1419 inverse-PCR
PWK9_BC1_gt_F	GGCCAATCGGAGTCTGAGGA	Validation for pNM1419 inverse-PCR
PWK9_BC1_gt_R	TGCTTTGTTCCCTGCCATGA	Validation for pNM1419 inverse-PCR
PWK6_BC1_SNP_F	ATACTCAAGGGCAGCCTTGC	Validation for pNM1419 inverse-PCR
pNM1419_iSeq_1st-PCR_F	TCGTCGGCAGCGTCAGATGTGTATAAGAGACAGATGGACGAGCTGTACAAGG	Amplicon-seq library 1st PCR
pNM1419_iSeq_1st-PCR_R	GTCTCGTGGGCTCGGAGATGTGTATAAGAGACAGGGATGGGATTCTTTAGGTCCTG	Amplicon-seq library 1st PCR
iSeq_2nd-PCR_N701	CAAGCAGAAGACGGCATACGAGATTCGCCTTAGTCTCGTGGGCTCGG	Amplicon-seq library 2nd PCR
iSeq_2nd-PCR_N702	CAAGCAGAAGACGGCATACGAGATCTAGTACGGTCTCGTGGGCTCGG	Amplicon-seq library 2nd PCR
iSeq_2nd-PCR_N703	CAAGCAGAAGACGGCATACGAGATTTCTGCCTGTCTCGTGGGCTCGG	Amplicon-seq library 2nd PCR
iSeq_2nd-PCR_N704	CAAGCAGAAGACGGCATACGAGATGCTCAGGAGTCTCGTGGGCTCGG	Amplicon-seq library 2nd PCR
iSeq_2nd-PCR_N705	CAAGCAGAAGACGGCATACGAGATAGGAGTCCGTCTCGTGGGCTCGG	Amplicon-seq library 2nd PCR
iSeq_2nd-PCR_N706	CAAGCAGAAGACGGCATACGAGATCATGCCTAGTCTCGTGGGCTCGG	Amplicon-seq library 2nd PCR
iSeq_2nd-PCR_S501	AATGATACGGCGACCACCGAGATCTACACTAGATCGCTCGTCGGCAGCGTC	Amplicon-seq library 2nd PCR
iSeq_2nd-PCR_S502	AATGATACGGCGACCACCGAGATCTACACCTCTCTATTCGTCGGCAGCGTC	Amplicon-seq library 2nd PCR
iSeq_2nd-PCR_S503	AATGATACGGCGACCACCGAGATCTACACTATCCTCTTCGTCGGCAGCGTC	Amplicon-seq library 2nd PCR
iSeq_2nd-PCR_S504	AATGATACGGCGACCACCGAGATCTACACAGAGTAGATCGTCGGCAGCGTC	Amplicon-seq library 2nd PCR
mES_BC_6-10_BC1_F	CGGTTGTTCGGTTTATTGTTTTTA	Validation for BC integration in mES BC lines
mES_BC_6-10_BC2_F	TGTGGGTTCGTACGTGATGA	Validation for BC integration in mES BC lines
mES_BC_6-10_BC3_F	GGGGGGTGGTTTGTGTCTT	Validation for BC integration in mES BC lines
mES_BC_6-10_BC4_F	GGATTTCGCCGTGGTGTTA	Validation for BC integration in mES BC lines
mES_BC_6-10_BC5_F	GTGTTGTGGCTGGGTGGT	Validation for BC integration in mES BC lines
mES_BC_6-10_BC6_F	GTATTTTGTCTGTTCGGTGTCG	Validation for BC integration in mES BC lines
mES_BC_6-10_BC7_F	GTGTTTTTTCGTTTGACGCTGT	Validation for BC integration in mES BC lines
mES_BC_6-10_BC8_F	GGTTTTACTGTCATGCTGGAGGT	Validation for BC integration in mES BC lines
mES_BC_6-10_BC9_F	GGTGTGTTGGTGCTTTTTGTTCT	Validation for BC integration in mES BC lines
mES_BC_6-10_BC10_F	GGTTTATAGTTGTTGTGCGCTGT	Validation for BC integration in mES BC lines
mES_BC_6-10_BC11_F	TGGTTTCGGCTGTTGGGA	Validation for BC integration in mES BC lines
mES_BC_6-10_BC12_F	GGGTTTTGTAGGGTTGCGTC	Validation for BC integration in mES BC lines
mES_BC_6-10_BC13_F	TGGTGTTGTTCGTGGTGG	Validation for BC integration in mES BC lines
mES_BC_6-10_BC14_F	GGTTTTATGTTTGCTCTTGGGTT	Validation for BC integration in mES BC lines
mES_BC_6-10_BC15_F	TTTTGGGGTTTCGTTTTTGTT	Validation for BC integration in mES BC lines
mES_BC_6-10_BC16_F	CGGTTTTGTTGGTTATGGTTTCTT	Validation for BC integration in mES BC lines
mES_BC_6-10_BC17_F	TGTTTTGGGTTGTTGGCTTTA	Validation for BC integration in mES BC lines
mES_BC_6-10_BC18_F	CGGTTTTTGTTTGTTTTTGTTTTT	Validation for BC integration in mES BC lines
mES_BC_6-10_BC19_F	CCGGTTATTTCTTTATGGCTTATTG	Validation for BC integration in mES BC lines
mES_BC_6-10_BC20_F	TTTCGCGTGTTTTTTTTTTTG	Validation for BC integration in mES BC lines
mES_BC_6-10_BC21_F	GGTTTCTGTTTTGATGGTGTTTT	Validation for BC integration in mES BC lines
mES_BC_6-10_BC22_F	ACCGGTGGTTGTAAACTTCTTTTATA	Validation for BC integration in mES BC lines
mESC_BC_13-25_BC1_F	GGTTTGGTGTCGAGCTGGA	Validation for BC integration in mES BC lines
mESC_BC_13-25_BC2_F	ACCGGTGTTGGTTTTAGTTTTAGAGT	Validation for BC integration in mES BC lines
mESC_BC_13-25_BC3_F	GGTGTGGTTTTATGGGTTTGAGT	Validation for BC integration in mES BC lines
mESC_BC_13-25_BC4_F	GGTTGGTTCTTTTTATGTTTGCG	Validation for BC integration in mES BC lines
mESC_BC_13-25_BC5_F	GTTTTTGTGTTTTTGTGGCGTT	Validation for BC integration in mES BC lines
mESC_BC_13-25_BC6_F	GGTTTTTGTTTTTGTGTCGCTTT	Validation for BC integration in mES BC lines
mESC_BC_13-25_BC7_F	CGGTTTCTGTTTTGATGGTGTTTT	Validation for BC integration in mES BC lines
mESC_BC_13-25_BC8_F	GGTTTTTTTTGGGTTGTTTCTGT	Validation for BC integration in mES BC lines
mESC_BC_13-25_BC9_F	CGGTGTTTTTTTTTTTCGTCGTAA	Validation for BC integration in mES BC lines
mESC_BC_13-25_BC10_F	CGGTTGTTCGGTTTATTGTTTTTA	Validation for BC integration in mES BC lines
mESC_BC_13-25_BC11_F	GGATTTCGCCGTGGTGTTA	Validation for BC integration in mES BC lines
pNM1419_3'ITR_R	CGCATGTGTTTTATCGGTCTG	Validation for BC integration in mES BC lines
Gusb-probe-5S	TCCTATCGGGATTCGAACAG	Southern blotting control probe
Gusb-probe-3A	GCCACAGACCACATCACAAC	Southern blotting control probe
GFP_SBlot_Probe_F	GCGACGTAAACGGCCACAAGTTCAGC	Southern blotting GFP probe
GFP_SBlot_Probe_R	CTGGGTGCTCAGGTAGTGGTTGTCG	Southern blotting GFP probe
Tg-sp-library_1st_F	TAACTTACGGAGTCGCTCTACGATGGACGAGCTGTACAAGGGCGGTG	Transgene-specific scRNA-seq library 1st PCR
Tg-sp-library_1st-2nd_R	AATGATACGGCGACCACCGAGATCTACACTCTTTCCCTACACGACGCTCTTCCGATCT	Transgene-specific scRNA-seq library 1st/2nd PCR
Tg-sp-library_2nd_F	CAAGCAGAAGACGGCATACGAGATCGGTCTCGGCATTCCTGCTGAACCGCTCTTCCGATCTNNNNNNNNNNNCACTAATGGT AACTTACGGAGTCGCTCTACG	Transgene-specific scRNA-seq library 2nd PCR
